# Atlantic to Pacific: Outbreak of bivalve transmissible neoplasia detected in hybridizing soft-shell clams and eDNA in Puget Sound

**DOI:** 10.1073/pnas.2611852123

**Published:** 2026-06-23

**Authors:** Sydney A. Weinandt, Zachary J. Child, Dorothy Lartey, Angel Santos, Holden Maxfield, Jordana K. Sevigny, Fiona E. S. Garrett, Peter D. Smith, Rachael M. Giersch, Samuel F. M. Hart, Lucas Rabins, Samuel Kaiser, Anna Boyar, Jan Newton, Jesse Kerr, Franchesca Perez, James L. Dimond, Michael J. Metzger

**Affiliations:** ^a^Pacific Northwest Research Institute, Seattle, WA 98122; ^b^ Department of Biology, University of Alabama at Birmingham, Birmingham, AL 35294; ^c^ Department of Biochemistry, University of Washington, Seattle, WA 98195; ^d^ Shannon Point Marine Center, Western Washington University, Anacortes, WA 98221; ^e^ Environmental Science and Management Department, Portland State University, Portland, OR 97201; ^f^ Colorado College, Colorado Springs, CO 80903; ^g^ Department of Ocean Sciences, University of California, Santa Cruz, CA 95064; ^h^ Institute of Molecular Biology, University of Oregon, Eugene, OR 97403; ^i^ Department of Genome Sciences, University of Washington, Seattle, WA 98195; ^j^Natural and Cultural Resources Department Shellfish Program, Tulalip Tribes of Washington, Tulalip, WA 98271; ^k^ Washington Ocean Acidification Center, University of Washington, Seattle, WA 98195; ^l^Prince Edward Island Department of Fisheries, Tourism, Sport and Culture, Prince Edward Island C1E 3J3, Canada; ^m^Natural Resources Department, Stillaguamish Tribe, Arlington, WA 98223

**Keywords:** transmissible cancer, disseminated neoplasia, bivalve transmissible neoplasia, marine infectious disease, ecology and evolution of infectious disease

## Abstract

Bivalve transmissible neoplasias (BTNs) are transmissible cancers that have arisen in multiple bivalve species, including Eastern soft-shell clam (*Mya arenaria*) populations on the East Coast of North America. Two sublineages of a single clone (termed MarBTN) currently circulate at low enzootic levels (1 to 5%) in New England, the United States, and Prince Edward Island, Canada, but MarBTN has not previously been observed in soft-shell clams on the West Coast. In 2022, we collected soft-shell clams in Puget Sound, Washington, and unexpectedly found MarBTN in two sites. Prevalence increased in subsequent years, surpassing 75% at both sites in 2024, while remaining undetectable in other populations, suggesting the early stages of a severe disease outbreak. Based on the presence of somatic transposon insertion sites found only in the USA-sublineage, the cancer was likely recently transplanted from New England. We also found that soft-shell clams in multiple Puget Sound locations are hybridizing populations of *M. arenaria* and *Mya japonica*. *M. japonica* may have decreased susceptibility to this cancer, though further sampling will be needed to confirm this hypothesis. We further developed a sensitive environmental DNA (eDNA) assay, targeting somatic mutations in the MarBTN mitogenome. Using this assay, we surveyed 51 sites throughout Puget Sound, detecting cancer at high levels at sites where MarBTN-positive clams were observed and in the surrounding area. These results identify a severe outbreak of transmissible cancer in a hybridizing population, due to transfer of disease from another ocean, and demonstrate the utility of eDNA methods to track BTN through the environment.

## Significance

We report a severe outbreak of transmissible cancer in soft-shell clams on the West Coast of North America (in Puget Sound, WA). We found high prevalence of disease in two locations, in contrast to the consistent low levels of cancer observed in East Coast populations, and we show that the outbreak arose from a transfer of infectious cells from the East Coast. Our development of sensitive methods to quantify the spread of cancer in seawater will be critical to future understanding of both local and world-wide transmission of this emerging class of infectious agent. Furthermore, identification of an acute outbreak in a hybridizing population provides a unique opportunity to observe host evolution in response to transmissible cancer in real time.

Most known cancers arise within an animal and remain within the same individual until the end of their life; however, there are a few rare types of horizontally transmitted cancers in which the cancer cells from one individual directly infect and engraft in other animals. Transmissible cancers were first found in dogs as canine transmissible venereal tumor disease (CTVT) (1, 2) and Tasmanian devils as devil facial tumor disease (DFTD) (3). These tumors are transmitted between individuals through physical contact, and in all cases exhibit genotypes that do not match their hosts but rather show clear clonal, transmissible lineages—a single lineage in dogs, and two independent lineages in devils (4). In many bivalve species, a lethal, leukemia-like disseminated neoplasia (DN) has been determined to be a bivalve transmissible neoplasia (BTN) (5–7), with at least 10 independent lineages observed affecting at least 10 different species (8–13).

BTN is characterized by the accumulation of neoplastic cells in the hemolymph, or circulatory fluid, of the bivalve, followed by dissemination into the solid tissues of the animal as the disease progresses until the death of the animal (14, 15). These cancer cells are rounded, nonadherent, and usually polyploid, in contrast to healthy bivalve hemocytes. BTN cells are capable of surviving in artificial sea water for weeks, indicating that the method of transmission is likely through the water column (16–18).

DN was first reported in mussels and oysters in 1969 (19, 20), and in Eastern soft-shell clams (*Mya arenaria*) on the East Coast of North America in the 1970s (21, 22). BTN is thought to be the causative agent of the severe outbreaks of DN that were reported in these populations that reached up to 90% prevalence, with severe population losses in the United States in the 1980s and in Prince Edward Island, Canada in the 2000s (23, 24). Recent analysis has shown that the current prevalence is stable and much lower (~1 to 5%), and there is evidence of evolution of protection from MarBTN progression in clams from the East Coast—approximately half of clams with MarBTN progress to high levels of cancer and die, as expected, but MarBTN does not progress in the other half, due to unknown mechanisms (25). All samples of DN from Eastern soft-shell clams on the East Coast that have been analyzed for genetic markers come from a single lineage of cancer (MarBTN), with two discrete sublineages in clams from the United States and from Prince Edward Island (PEI) that are estimated to have diverged ~300 y ago (26).

The current populations of soft-shell clams on the West Coast of North America, and in Washington State in particular, are not native. Eastern soft-shell clams (*M. arenaria*) were likely intentionally introduced from the Atlantic populations of clams in the 1870s (27, 28). Additionally, Japanese soft-shell clams from the western Pacific (*Mya japonica*) have been reported in this area (29). While the two species cannot be clearly distinguished based on morphology (*SI Appendix*, Fig. S1), they have been repeatedly reported to have distinct mitochondrial sequences and were not previously reported to hybridize (30).

Previously, MarBTN had only been reported in soft-shell clams on the Atlantic coast of North America, so these circumstances appeared to provide the opportunity to study an entirely naive population of soft-shell clams on the West Coast, compared with exposed individuals on the East Coast. However, when we sampled 47 soft-shell clams from Triangle Cove, WA in 2022, we unexpectedly found MarBTN at a prevalence of 45%. These findings prompted us to investigate the presence and spread of MarBTN in the Puget Sound, WA.

Environmental DNA (eDNA) is increasingly used in marine and aquatic conservation efforts, especially in detecting invasive species and pathogens (31–36). In marine systems, eDNA sampling entails filtering seawater to capture DNA present in the water column, which is then extracted and amplified for analysis, allowing for insights into community makeup or identification of specific species. When used together with highly sensitive assays such as qPCR or ddPCR, eDNA samples can be used to detect trace amounts of target DNA (37). While not eliminating the need for traditional sampling methods, eDNA sampling is an effective tool in monitoring efforts (31). Previous studies have demonstrated the ability of eDNA analysis to detect a variety of marine pathogens and parasites, such as MSX, perkinsosis (38), **Vibrio* spp.* (39), and abalone withering syndrome (35). Previous studies from our group investigated the use of eDNA for the detection of transmissible cancers, showing detection of BTN-specific DNA in tank water of clams in aquaria (17, 25). Here we build on those eDNA protocols and develop extraction and amplification methods that enable sensitive detection of MarBTN in wild samples.

First, we report the identification of an outbreak of MarBTN on the West Coast of the United States and test whether it represents spread of MarBTN from the East Coast, or a new lineage. We also genotyped soft-shell clams at a nuclear and mitochondrial locus and found that both *M. arenaria* and *M. japonica* are present at multiple locations throughout Puget Sound and that there is significant evidence of hybridization between the species. Second, to determine the extent of the spread of this outbreak and to develop tools for disease monitoring, we use both animal collection and eDNA surveys to observe the disease in the environment throughout the Puget Sound.

## Materials and Methods

### Clam Collection and Sampling.

**Fig. 1.**
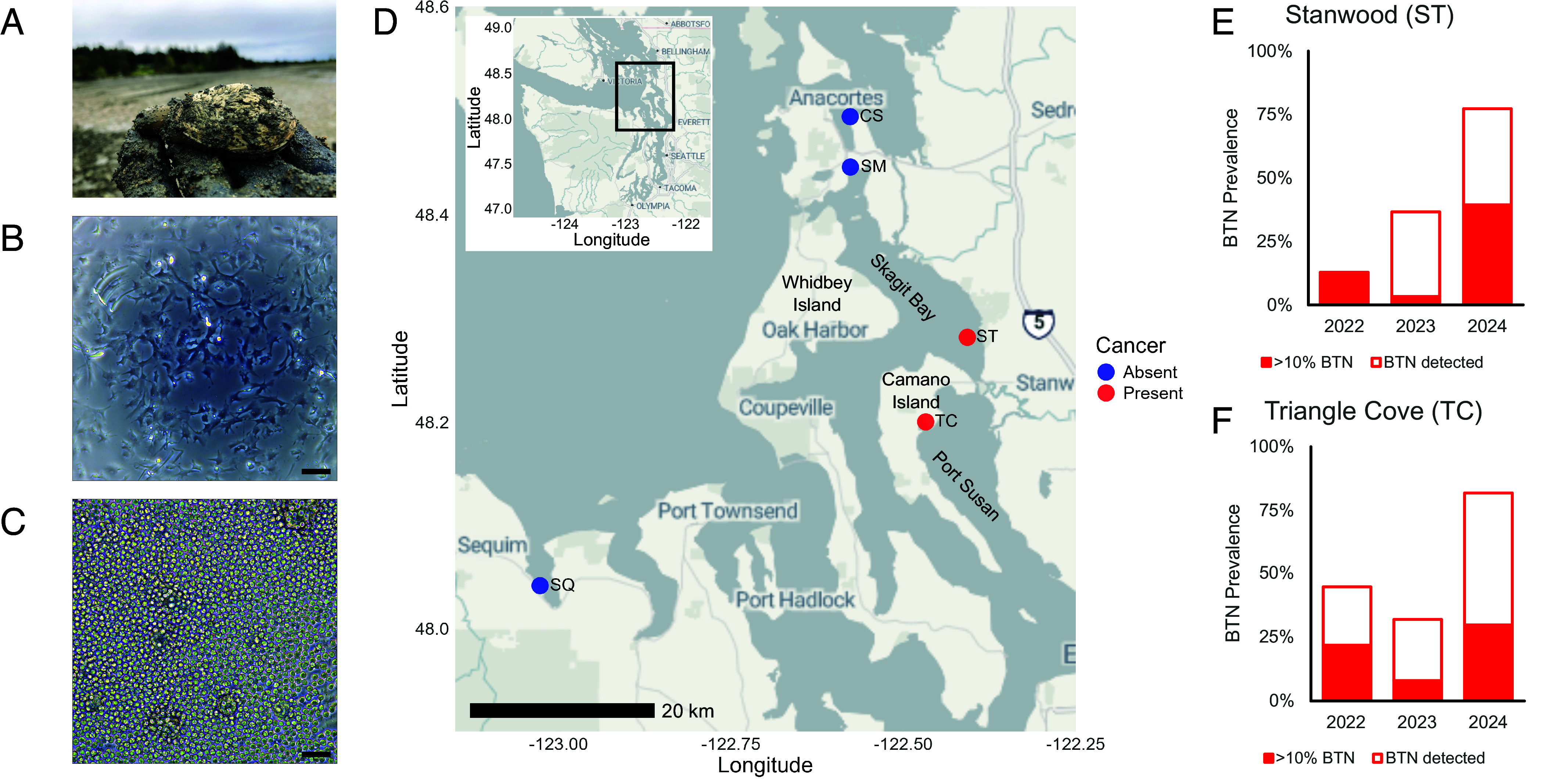
Detection of BTN in soft-shell clams (*Mya sp.*) in Puget Sound, Washington State, the United States. (*A*) Representative soft-shell clam (*Mya sp.*) from Triangle Cove, Washington, the United States, collected in 2022 is shown. *M. arenaria* and *M. japonica* cannot be readily distinguished morphologically. (*B*) Representative healthy hemocytes from a clam from Crandall Spit, WA (WACS-B2) and (*C*) BTN from a clam from Triangle Cove, WA (WATC-D5) show very different morphology (flattened, adherent healthy hemocytes with pseudopodia and rounded, refractive, nonadherent MarBTN cells). (Scale bar, 50 µm.) (*D*) Map shows the locations of clam collections with colors denoting whether BTN is present (red) or absent (blue). Inset map shows the Washington State coastline including all of Puget Sound, with the zoomed in region marked by a black rectangle. Samples were collected from 2022–2024. BTN was detected in clams at Triangle Cove (TC) and in Stanwood (ST) 2022–2024, and prevalence at each collection is shown in (*E* and *F*). Filled bars show fraction of collected clams with >10% cancer in the hemolymph and total filled and unfilled bar height reflects total prevalence of clams with any detectable BTN. BTN was never detected in clams from other locations at any collection 2022–2024. Specific prevalence of BTN at each collection is shown in Table 1. Maps were generated using R packages ggplot (40), ggmap (41), and dplyr (42). Map style was generated by StadiaMaps, using map type “alidade_smooth.”

Adult soft-shell clams were collected from five locations in Puget Sound, Washington (Triangle Cove, Crandall Spit, near Stanwood, Similk Bay, and Sequim Bay). From each site, between 3 to 66 soft-shell clams were collected once each year from 2022 to 2024 (Table 1 and *SI Appendix*, Fig. S1). Animals were collected from the intertidal zone during low tides with a shovel for all sites except near Stanwood, at which clams were collected by a commercial supplier. All animals were measured, and 0.5 to 1 mL of hemolymph was taken from the pericardial region using a 0.5 inch 26-gauge needle (Fisher Scientific) fitted on a 3 mL syringe (Fisher Scientific) (17). Approximately 20 to 50 µL of hemolymph was placed in a flat-bottom 96-well plate (Fisher Scientific) and incubated at 4 °C for approximately 1 h before screening for the morphologically distinct cancer cells on an inverted phase-contrast microscope (Leica DMi1) (Fig. 1). The remaining hemolymph was placed into a 1.7 mL microtube (Genesee Scientific), and spun down in a centrifuge (MilliporeSigma) at 4 °C at 1,000×*g*. The supernatant was removed, and the cell pellets were kept at −80 °C until DNA extraction. Hemolymph DNA was extracted using the Monarch Genomic DNA Purification kit (New England Biolabs).

**Table 1.** MarBTN prevalence in clams collected in Puget Sound, WA, the United States, from 2022 to 2024

Location	Code	Coordinates	Collection date	Clams tested	MarBTN positive	Prev.	MarBTN >10%	Prev. >10%	*M. arenaria* *EF1α* *	*M. arenaria* *mtCOI*
Triangle Cove	TC	48.20013, −122.46748	4/20/2022	60	27	45%	14	23%	47	58
			5/10/2023	50	16	32%	4	8%	34	45
			7/8/2024	27	22	81%	8	30%	24	25
Stanwood	ST	48.24112, −122.37181	9/29/2022	39	5	13%	5	13%	39	39
			8/2/2023	30	11	37%	1	3%	30	30
			7/5/2024	66	51	77%	26	39%	66	65
Crandall Spit	CS	48.2941, −122.574528	5/4/2022	48	0	0%	0	0%	7	7
			6/6/2023	34	0	0%	0	0%	2	2
			6/20/2024	27	0	0%	0	0%	0	0
Similk Bay	SM	48.445834, −122.57675	7/6/2023	37	0	0%	0	0%	9	10
			7/22/2024	15	0	0%	0	0%	10	9
Sequim Bay	SQ	48.0319, −123.01985	8/30/2023	3	0	0%	0	0%	0	0
			2/6/2024	13	0	0%	0	0%	0	0

^*^Number of clams genotyped as homozygous for *M. arenaria* alleles at the *EF1α* locus. This excludes homozygous *M. japonica* and *japonica*/*arenaria* heterozygotes.

### MarBTN qPCR Diagnostic Targeting a Nuclear Marker.

Allele-specific qPCR assays were conducted to quantify the neoplastic and host genomic DNA in the hemolymph. Two pairs of primers were used to target a nuclear locus, with the cancer-specific pair targeting a somatic insertion of the LTR-retrotransposon *Steamer* near the *N1N2* gene and the “universal” clam pair targeting a conserved region of the *N1N2* ORF nearby (17, 25). As done previously, the plasmid pCR-SteamerLTR-N1N2, which contains a single copy of the locus from a cancer cell, and which therefore contains the target sequence for both cancer and control primers, was used for the standard curve. Primers are listed in *SI Appendix*, Table S2 and standard curves shown in *SI Appendix*, Fig. S2. 5 µL of the plasmid miniprep was linearized using 0.5 µL of NotI-HF (New England Biolabs) for 2 h at 37 °C in a 10 µL reaction, and heat-inactivated at 65 °C for 20 min. DNA concentration was determined using a Qubit Fluorometer (Invitrogen, ThermoFisher Scientific), absolute plasmid copy number per µL was calculated based on the known size of the full plasmid (4,403 bp), and stocks were diluted to 1 × 10^9^ copies/µL with NEB Elution Buffer (New England Biolabs). Standard curves were prepared through further dilution to ten-fold reductions between 1 × 10^7^ copies per reaction to 10 copies per reaction. Reactions were run using PowerUp SYBR Green Master Mix for qPCR (Applied Biosystems, ThermoFisher Scientific) on a StepOnePlus Real-Time PCR System (Applied Biosystems, ThermoFisher Scientific) with a holding stage at 95 °C for 2 min, two step cycling at 95 °C for 15 s and 60 °C for 30 s for 40 cycles, followed by the melt curve stage (2 µL of DNA per reaction). All samples were run in triplicate and values were averaged. Samples were considered positive if cancer amplification was detected in all three wells, with an average of at least one copy per reaction. Using these values, we used the ratio (R) of the total copies of the cancer-specific allele to the total copies of the *N1N2* locus present to estimate the fraction of MarBTN cells in the clam hemolymph. The estimated fraction of cancer cells in the hemolymph is R/(1−R) to adjust for the difference in ploidy of the diploid normal cells to the polyploid MarBTN cancer cells, which are tetraploid at this locus in the USA sublineage, with the somatic insertion site present in 2 of 4 copies, based on genomic analysis (26), likely due to somatic insertion of the retrotransposon before a duplication of that region. The full derivation of this formula is found in the supplementary text of Giersch et al. (25).

### Retrotransposon Insertion Site-Specific PCR.

To compare the genotype of MarBTN on the West Coast of the United States to those from the East Coast of the United States and Canada, we used 17 different reverse primers that target specific insertion sites of the LTR-retrotransposon, *Steamer,* paired with a single forward primer that matches the end of the *Steamer* LTR (ClamLTR-F2). Previously reported primers were identified through inverse-PCR cloning strategies using genomic DNA of MarBTN samples (6, 43), and new primers were designed based on analysis of *Steamer* insertion sites from whole genome sequencing analysis (26). Briefly, new primers were selected within the 200 bp downstream of insertion sites, excluding sites which had multiple blastn hits in the *M. arenaria* reference genome (GCF_026914265.1), and selecting only one locus per chromosome, starting with chromosome 1. Primers are listed in *SI Appendix*, Table S2. For each 25 µL reaction, 2.5 µL of DNA was used. Thermocycler (MiniAmp Plus, AppliedBiosystems) conditions were 95 °C for 5 min, 35 cycles of 95 °C for 30 s, 50 °C for 30 s, and 72 °C for 30 s, followed by 72 °C for 5 min. The amplified DNA products were analyzed using 2% agarose gel electrophoresis with 10 µL of product per well.

### Genotyping of *M. arenaria* and *M. japonica*.

We amplified one nuclear locus, elongation factor 1 alpha (*EF1α*) and one mitochondrial locus, cytochrome c oxidase I (*mtCOI*). Genomic DNA from clams was amplified using primers listed in *SI Appendix*, Table S2. For *EF1α*, each 25 µL reaction used 2.5 µL of DNA. Thermocycler (Applied Biosystems) conditions were: 95 °C for 5 min, 35 cycles of 95 °C for 30 s, 52 °C for 30 s, and 72 °C for 20 s, followed by 72 °C for 5 min. For *mtCOI*, each 25 µL reaction used 1 µL of DNA. Thermocycler (Applied Biosystems) conditions were: 95 °C for 5 min, 35 cycles of 95 °C for 30 s, 55 °C for 30 s, and 72 °C for 30 s, followed by 72 °C for 5 min. For each amplicon, a species-specific restriction site was identified (HaeIII for *mtCOI* and EagI-HF for *EF1α,* New England Biolabs), and was used to genotype all soft-shell clams tested in this study. For each PCR, a 10 µL digest was made using 5 µL of the PCR, 1 µL 10× cutsmart buffer (NEB), 3.7 to 3.875 µL of molecular biology grade water, and 0.3 µL HaeIII for 4 h at 37 °C or 0.125 µL EagI-HF for 2 h at 37 °C. For *mtCOI*, running the digested PCR product on a 1 to 1.5% agarose gel could distinguish amplicons that came from *M. arenaria* (255 and 457 bp bands) or *M. japonica* (intact 712 bp band). For *EF1α*, the digest was able to distinguish homozygous *M. arenaria* (intact 334 bp band), homozygous *M. japonica* (120 and 214 bp bands), and clams heterozygous at that locus (120, 214, and 334 bp bands). The validity of this assay was confirmed by Sanger sequencing of both loci from all clams from Triangle Cove and Crandall Spit in 2022 (Azenta). MarBTN itself is *M. arenaria*, so in clams with high levels of BTN, this could interfere with detection of *M. japonica* sequence from the host using the diagnostic digests above. Therefore, in any sample with >10% MarBTN (and any other samples with inconclusive results), we also used primers that specifically only amplify *M. japonica EF1α* and *mtCOI* to determine whether the host genotype includes *M. japonica* sequence that was masked by the MarBTN DNA. For clams with BTN that were positive for *M. japonica EF1α*, deep sequencing of the PCR product at >5,000× coverage (Plasmidsaurus) was used to determine whether the host was homozygous or heterozygous (*SI Appendix*, Fig. S3). DNA samples were quantified with a NanoDrop (Thermo Scientific), and aliquoted into a standardized dilution of 10 ng/µL. Each 25 µL reaction used 1 µL of diluted DNA. Thermocycler (Applied Biosystems, ThermoFisher Scientific) conditions for *M. japonica*-specific *EF1α* were: 95 °C for 5 min, 35 cycles of 95 °C for 30 s, 60 °C for 30 s, and 72 °C for 30 s, followed by 72 °C for 5 min. Annealing temperature was raised to 63 °C for *M. japonica-*specific *mtCOI* PCRs. All results are shown in *SI Appendix*, Table S1.

Maximum likelihood phylogenetic trees were made with Phyml 3.0 (44). The reference *EF1α* sequence from *M. japonica* was generated by using the Geneious assembler to map whole genome DNA short read sequence (SRR27229027) from a *M. japonica* sample from Qingdao, China (BioSample: SAMN38816529) to the *EF1α* gene in the *M. arenaria* reference, GCF_026914265.1 (26). All sequences (accession numbers for *mtCOI*: PZ222001-PZ222017; and *EF1α*: PZ233404- PZ233407) are available on GenBank.

Hardy–Weinberg equilibrium (HWE) and Linkage Disequilibrium (LD) were tested using Monte Carlo tests: chi^2^ tests with Monte Carlo simulation to derive the *P* value, in R using chisq.test (obs, p = p_exp, simulate.p.value = TRUE, B = 1e6), where obs was the observed counts and p _ exp were the expected genotype counts based on observed allele frequencies (for HWE only the single diploid *EF1α* locus was used and for LD both the diploid *EF1α* and the uniparentally inherited haploid *mtCOI* loci were used).

### Field Seawater eDNA Collection, Filtration, and DNA Extraction.

#### Shore sampling.

For collection, filtration, and extraction of eDNA from shore, seawater was collected by hand at low tide from each collection site into 3 × 500 mL separate autoclaved polypropylene laboratory bottles (Fisher Scientific). Samples were held in a cooler on ice until return to the lab 2 to 4 h later, after which they were frozen at −30 °C until filtration using a 0.45 μm cellulose nitrate filter (Fisher Scientific) and an autoclaved, bench-top vacuum filtration apparatus (Fisher Scientific). Each 500 mL biological replicate was filtered and extracted separately. The unused outer ring of each filter was trimmed off, and the filter was cut into 6 pieces to facilitate contact with the beads during extraction. Filters were stored at −80 °C until DNA extraction with DNeasy PowerSoil Pro Kits (Qiagen). See *SI Appendix*, Text S1 for extended protocol.

#### Sampling by ship.

Water samples were collected from several cruises conducted in 2024: a collection throughout the Skagit Bay with Western Washington University, a collection in Tulalip Bay with the Tulalip Tribes, and a larger survey of Puget Sound conducted on the *R/V Rachel Carson* with the Washington Ocean Acidification Center (WOAC). Due to conditions onboard a ship and protocols of the WOAC, modifications to eDNA protocols are as follows:

For Skagit Bay and the detailed collections around Triangle Cove, seawater samples were collected from one meter off the seafloor with a handheld Niskin bottle. Proper depth was determined by attaching a one-meter line with a weight on the bottom end to the Niskin bottle. Seawater was collected from each site into 3 × 500 mL separate autoclaved polypropylene laboratory bottles (Fisher Scientific) in Skagit Bay. For the analysis of local spread around Triangle Cove, 1 × 500 mL bottle was collected at each site at two different times in the same day. Collection bottles and the Niskin bottle were bleached and rinsed in between sampling stations. Each sample was immediately filtered through a 0.45 μm cellulose nitrate filter using Nalgene Single Use Analytical Filter Funnels (Fisher Scientific). Filters were then placed into single-use coin envelopes (Eupako/Amazon), subsequently sealed in a mylar ziplock bag (QQ Studio/Amazon) with a silica gel desiccant packet (Dry&Dry/Amazon), and held on ice for short-term preservation. Upon return to the laboratory, filters were held at −80 °C until extraction with DNeasy PowerSoil Pro Kits (Qiagen).

For Tulalip Bay, 3 × 500 mL seawater samples were collected from one meter off the seafloor with a handheld Niskin bottle as for Skagit Bay collections. The Niskin bottle was thoroughly rinsed with seawater upon arrival at the following sampling station. As with samples collected from shore, water samples were held in a cooler on ice until return to the lab 2 to 4 h later, after which they were frozen at −30 °C until filtration through a 0.45 μm cellulose nitrate membrane (Fisher Scientific) with an autoclaved, bench-top vacuum filtration apparatus (Fisher Scientific), and filters were held at −80 °C until extraction with DNeasy PowerSoil Pro Kits (Qiagen), as described above.

##### For WOAC cruises on the R/V Rachel Carson.

Seawater samples were collected by a Niskin rosette attached to the ship’s conductivity, temperature, and depth sensor (CTD). Three Niskin bottles were used for each site, with one closed at the bottom of the cast, one in the middle, and one at the surface of the water. For each bottle, exact closure depth was recorded. 500 mL of seawater was collected from each Niskin and transferred into a 500 mL autoclaved polypropylene laboratory bottle (Fisher Scientific). Collection bottles were bleached between sites to prevent cross-contamination of samples. Each sample was immediately filtered through a 0.45 μm cellulose nitrate filter using Nalgene Single Use Analytical Filter Funnels (Fisher Scientific). Filters were then placed into 900 μL of Longmire’s solution, a lysis buffer which preserved samples until DNA extraction (45, 46), and eDNA was later extracted with a phenol:chloroform:isoamyl alcohol protocol, modified from (47). Modifications to the published protocol, as well as a full list of reagents and devices used for eDNA collection and analysis, are detailed in *SI Appendix*, Text S1.

For all samples, the extracted eDNA yield was quantified with a NanoDrop (Thermo Scientific) and samples were stored in 1.7 mL microtubes (Genesee Scientific) at −30 °C until qPCR analysis. Biological replicates were extracted and analyzed by qPCR separately (each in triplicate qPCRs) before averaging. For details of all eDNA collections, see *SI Appendix*, Table S3. A detailed protocol for eDNA extraction can be found in *SI Appendix*, Text S1. In order to directly compare the eDNA yields from these variations in eDNA extraction, we collected three sets of triplicate samples from one site in south Skagit Bay at which MarBTN had been previously detected (Big Ditch Trail, 48.271592, −122.40523). For each of the three processing and extraction methods, we extracted one set of triplicate water samples and compared the resulting qPCR values after amplification with all three primer sets (MarBTN, *M. arenaria*, and *M. japonica*).

### qPCR of eDNA.

To quantify the presence of neoplastic DNA, *M. arenaria* DNA, and *M. japonica* DNA in an eDNA sample, allele-specific qPCR primers targeting somatic single nucleotide variants (SNVs) in mitochondrial DNA were created. Based on previous genomic analysis of MarBTN (26), we identified loci on the mitogenome with two closely spaced SNVs found only in the USA sublineage of MarBTN (which were therefore somatic mutations that would be unique markers of cancer DNA). We designed qPCR primers (MarBTN-mt8807F1/MarBTN-mt8843R2) to specifically target these MarBTN-USA mutations (T8807G and C8843T), and we designed control primers (MarBTN-mt8807NORMF1/MarBTN-mt8843NORMR1 and Mjp-mt8807NORMF1/ Mjp-mt8843NORMR1) to amplify the same region, excluding the site of the SNVs. Primers are listed in *SI Appendix*, Table S2. Two standard plasmids (pCR-MarBTNmt88 and pCR-Mjpmt88) were cloned with the Invitrogen Zero Blunt Topo PCR Cloning Kit (Invitrogen, ThermoFisher Scientific), utilizing the control primers to amplify the mitochondrial DNA of MarBTN cells and *M. japonica* cells, respectively. The plasmids were linearized using 0.5 µL of NotI-HF (New England Biolabs) for 2 h at 37 °C in 10 µL reactions, and heat-inactivated at 65 °C for 20 min. DNA concentrations were determined using a Qubit fluorometer (Invitrogen, ThermoFisher Scientific), absolute copy numbers per µL were calculated based on total plasmid size (4,041 bp), and stocks were diluted to 1 × 10^9^ copies/µL with NEB Elution Buffer (New England Biolabs). Standard curves were prepared through further dilution to ten-fold reductions between 1 × 10^7^ copies per reaction to 10 copies per reaction. For eDNA, 20 µL reactions were run with 2 µL of extracted DNA (using a higher volume of extracted DNA or a lower reaction volume led to errors due to PCR inhibitors in some samples). We used PowerUp SYBR Green Master Mix for qPCR (Applied Biosystems, ThermoFisher Scientific) on a StepOnePlus Real-Time PCR System (Applied Biosystems, ThermoFisher Scientific), with a holding stage at 95 °C for 2 min, 40 cycles of 95 °C for 15 s and 60 °C for 30 s, followed by the melt curve stage. All samples were run in triplicate and averaged. Samples were considered positive if amplification was detected in all triplicate wells above 1 copy per reaction. Absolute quantification measured as copies per reaction were normalized to copies per mL of seawater, assuming 100% efficiency of eDNA extraction from water samples

## Results

### Identification and Tracking of an Outbreak of BTN in Soft-Shell Clams in Triangle Cove and South Skagit Bay, Washington, the United States.

In 2022, when we sampled hemolymph from clams from Triangle Cove, Puget Sound, WA, we unexpectedly found that many clams (27 of 60; 45%) had abnormal cell morphology, consistent with DN (Fig. 1). We confirmed that the DN in these clams was MarBTN using qPCR primers that amplify a characteristic retrotransposon insertion site in the clam genome that is specific to this cancer lineage (17, 26). We then tested a second location close to Triangle Cove (Stanwood, in south Skagit Bay) and found cases of MarBTN there as well (5 of 39; 13%). In 2022 and later years, we sampled more broadly, collecting and diagnosing animals from 5 different beaches in Puget Sound, and found increasing prevalence of MarBTN at the two positive sites in 2023 and 2024 (Fig. 1 and Table 1), with high severity of infection in many cases (Fig. 1 and *SI Appendix*, Fig. S5). In 2023 at Triangle Cove, we found 16 out of 50 animals (32%) to be positive by qPCR, and in 2024 we found 22 out of 27 animals (81%) to be positive, with 30% having more than 10% cancer cells in their hemolymph, indicating severe disease (Table 1). In 2023 at Stanwood, we found 11 out of 30 animals (37%) to be positive, and in 2024 we found 51 out of 66 animals (77%) to be positive, and 39% had more than 10% cancer cells in their hemolymph (Table 1). At the remaining 3 locations, Crandall Spit, Similk Bay, and Sequim Bay, we did not observe any abnormal cell morphology or positive amplification of cancer DNA from any animals sampled in 2022, 2023, or 2024 (Table 1).

### Molecular Analysis Suggests Cancer in Puget Sound Clams Came From the East Coast of the United States.

**Fig. 2.**
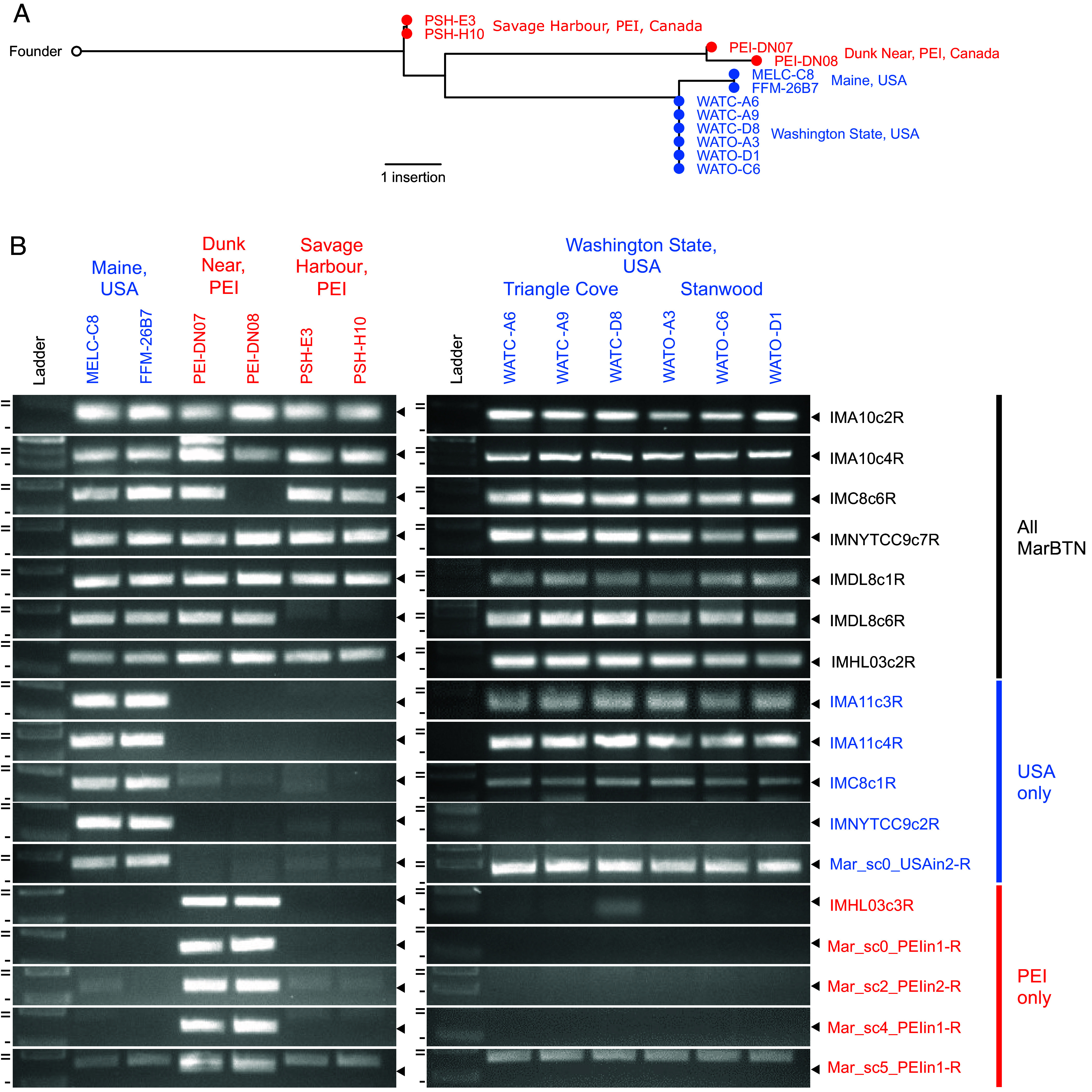
Transposon insertions show BTN in soft-shell clams in Puget Sound is from the USA sublineage of MarBTN. Multiple PCR primers diagnostic for specific insertion sites of the retrotransposon *Steamer* were used to amplify MarBTN DNA from clams in Puget Sound (three from Triangle Cove, WATC, and three from Stanwood, WATO) as well as from MarBTN DNA from clams known to carry the previously known USA sublineage (MELC and FFM), the previously known PEI sublineage (PEI), and previously undescribed, more divergent samples collected from Savage Harbour, PEI (PSH). Of these 17 primers, seven target *Steamer* insertion sites previously identified to be present in both PEI and USA MarBTN samples, five were specific to MarBTN from USA, and five were specific to MarBTN from PEI (*SI Appendix*, Table S2). (*A*) A neighbor-joining phylogenetic tree was made using the presence or absence of each insertion site (using the ape package in R), rooted with a hypothetical Founder clam (open circle) with none of these insertions. Scale bar shows the genetic distance corresponding to one insertion site. (*B*) Gels show the products of each diagnostic PCR. Labels on the *Right* note the reverse primer used and whether the *Steamer* insertion sites have been previously observed in both USA and PEI sublineages (black), or were unique to the United States (blue) or PEI (red) in previous studies (6, 26, 43). Black triangles on the *Right* of each gel mark the expected products. All products are 120 to 170 bp (*SI Appendix*, Table S2), and the 100 and 200 bp ladder bands are marked (− and =, respectively). In the case of Mar_sc5_PEIin1-R, a nonspecific PCR product can be observed in all samples that is larger than the expected product (which is only present in PEI-DN07 and PEI-DN08).

The cancer cells in Washington State clams were detected using PCR primers that amplify a *Steamer* retrotransposon insertion found in MarBTN from Eastern soft-shell clams on the East Coast of North America (17, 25), so the cancer detected in Puget Sound soft-shell clams is likely from this same lineage, which arose from a single clam several hundred years ago (26). In order to confirm this and to determine whether the cancer was transferred to the West Coast from one of the existing sublineages of MarBTN (USA or PEI) or whether it diverged prior to the split of those two sublineages, we used PCR primers that are diagnostic for specific *Steamer* integration sites that were identified in MarBTN samples from both sublineages or were specifically found in only one of the two sublineages (6, 26, 43). As a comparison, we tested two samples of MarBTN from the East Coast of the United States and four from PEI clams [two collected previously (6) and two recently collected]. All of the DNA samples from both East Coast MarBTN and West Coast MarBTN amplified with the primers that target insertions previously known to have occurred prior to the USA/PEI split, with the exception that the two newer samples from Savage Harbour, PEI (PSH-E3 and PSH-H10) did not amplify with primer IMDL8c6R (Fig. 2). There was also one primer pair amplifying an early insertion present in both lineages that did not amplify with sample PEI-DN08, showing that there is some variability within the sublineage, likely due to deletion of the region containing the insertion. The PEI-specific primers amplified the targets in the two samples previously collected from the Dunk River Estuary, PEI (PEI-DN07 and PEI-DN08), but did not amplify in West Coast samples or in the two newer samples from Savage Harbour, suggesting that these newer PEI samples may reflect a new sublineage that branched off from the ancestral lineage either near the time of the previously reported USA/PEI split or before. The USA-specific primers amplified all of the East and West Coast USA samples except for primer IMNYTCC9c2R, which did not amplify in any of the West Coast samples (Fig. 2). We additionally amplified and Sanger sequenced the mitochondrial cytochrome c oxidase I (*mtCOI*) locus from these samples, and confirm that the sequence is identical to that previously found in the USA sublineage of MarBTN, without the SNV specific to PEI MarBTN (6). Therefore, the MarBTN in Puget Sound, on the West Coast of the United States, closely matches known samples from the East Coast USA sublineage, and was likely recently transported from New England to the location of this outbreak.

### Detection of Hybridizing Populations of *M. arenaria* and *M. japonica* Soft-Shell Clams in Puget Sound, Washington.

**Fig. 3.**
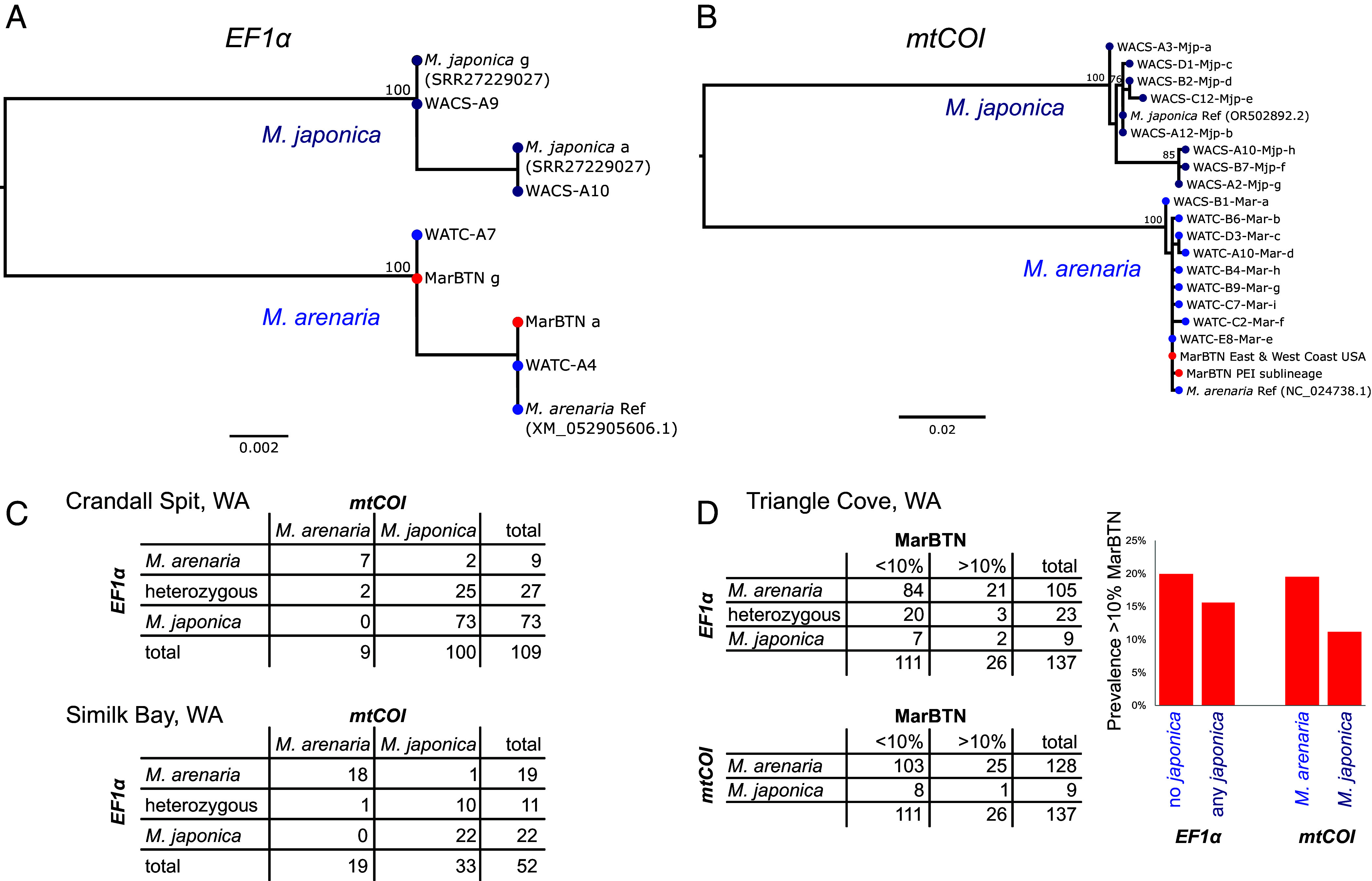
Identification of hybridizing populations of *M. arenaria* and *M. japonica* in Puget Sound. We sequenced a fragment of the nuclear gene, *EF1α* (*A*), and the mitochondrial cytochrome c oxidase I, *mtCOI* (*B*), from hemolymph DNA from all clams from the 2022 collections from Crandall Spit, WA (n = 48, MarBTN negative), and Triangle Cove, WA (n = 32 MarBTN negative, 26 MarBTN positive), and made maximum likelihood phylogenetic trees with a single representative of each unique sequenced allele, together with known representative reference sequences for *M. arenaria* (26, 48) and *M. japonica* (30). Based on the sequence data, we used a diagnostic species-specific restriction site to genotype all clam samples from all collections (*SI Appendix*, Table S1 and Fig. S3). We compared the genotypes of each clam at nuclear and mitochondrial markers showing results for two sites where both species are present and MarBTN is absent (*C*), showing evidence of hybridization between species. Hardy–Weinberg equilibrium can be rejected at both collection sites for *EF1α* (*P* < 0.05 for both) and there is significant linkage disequilibrium between *EF1α* and *mtCOI* (*P* < 1 × 10^−6^). We also compared prevalence of severe MarBTN (>10%) with presence of *M. japonica* DNA in each clam at both loci, in clams from Triangle Cove. (*D*) The table shows the numbers of clams with severe MarBTN (>10%) compared to number of negative and low positive clams (<10%). Since the MarBTN is *M. arenaria*, in addition to the diagnostic digests, we used a *M. japonica*-specific PCR to test for the presence of any *M. japonica EF1α* and *mtCOI* DNA in clams with >10% MarBTN, and >5,000× coverage sequencing of *EF1α* amplicons from *M. japonica*-positive, MarBTN-positive clams to determine whether they had one or two *M. japonica* alleles (*SI Appendix*, Fig. S3). Only one animal collected from Stanwood had *M. japonica mtCOI and M. arenaria EF1α,* and all other individuals were *M. arenaria* at both loci.

While soft-shell clams in Washington state have been primarily reported to be *M. arenaria*, there have been some reports of *M. japonica* in the area (29). We therefore genotyped all soft-shell clams sampled, and found that both species are present in Puget Sound, with some populations (like Stanwood and Triangle Cove) dominated by *M. arenaria*, and other populations dominated by *M. japonica* or with a more even mix of the species (Table 1 and Fig. 3). There were no previous reports of hybridization, but previous studies only sequenced mitochondrial loci with species-specific differences (29, 30), so would be unable to detect any hybridization or introgression. We sequenced both a mitochondrial locus (*mtCOI*) and a nuclear locus (*EF1α*) and found widespread evidence that hybridization has occurred, with many examples of clams heterozygous for the *M. arenaria* and *M. japonica* alleles at the nuclear locus and examples of clams with discordant genotypes in the nuclear and mitochondrial markers (Fig. 3). We tested the clams collected from two sites with both species but without MarBTN (Crandall Spit and Similk), and found that alleles at the single nuclear locus are not in Hardy–Weinberg equilibrium (Monte Carlo test, *P* < 0.05 for clams from both collection sites). We also found that there is significant linkage disequilibrium between the nuclear and mitochondrial loci (Monte Carlo test, *P* < 1 × 10^−6^), suggesting that the species are still distinct and that there is still at least some hybridization barrier (Fig. 3*C*).

With these results we can confirm that MarBTN can infect soft-shell clams genotyped as both *M. arenaria* and *M. japonica* (using *EF1α* and *mtCOI* loci*),* although *M. arenaria* may be more susceptible (Fig. 3*D*). Only a single clam with >10% MarBTN was identified as homozygous *M. japonica* at the nuclear locus and *M. japonica* at the mitochondrial locus (WATC-G12), and since we only genotyped a single nuclear locus in a mixed population it is possible that this animal is not fully *M. japonica*. It is notable that the outbreak of MarBTN was identified in two populations with high proportions of *M. arenaria*. In samples from Triangle Cove, we found a trend toward higher MarBTN prevalence in animals with *M. arenaria* DNA (Fig. 3*D*). This trend is present whether looking at either nuclear or mitochondrial loci separately or together, but the trends are not statistically significant.

### Sensitive Mitochondrial qPCR on eDNA From Triangle Cove, Washington, Shows Local Spread of Cancer Cells in Seawater.

**Fig. 4.**
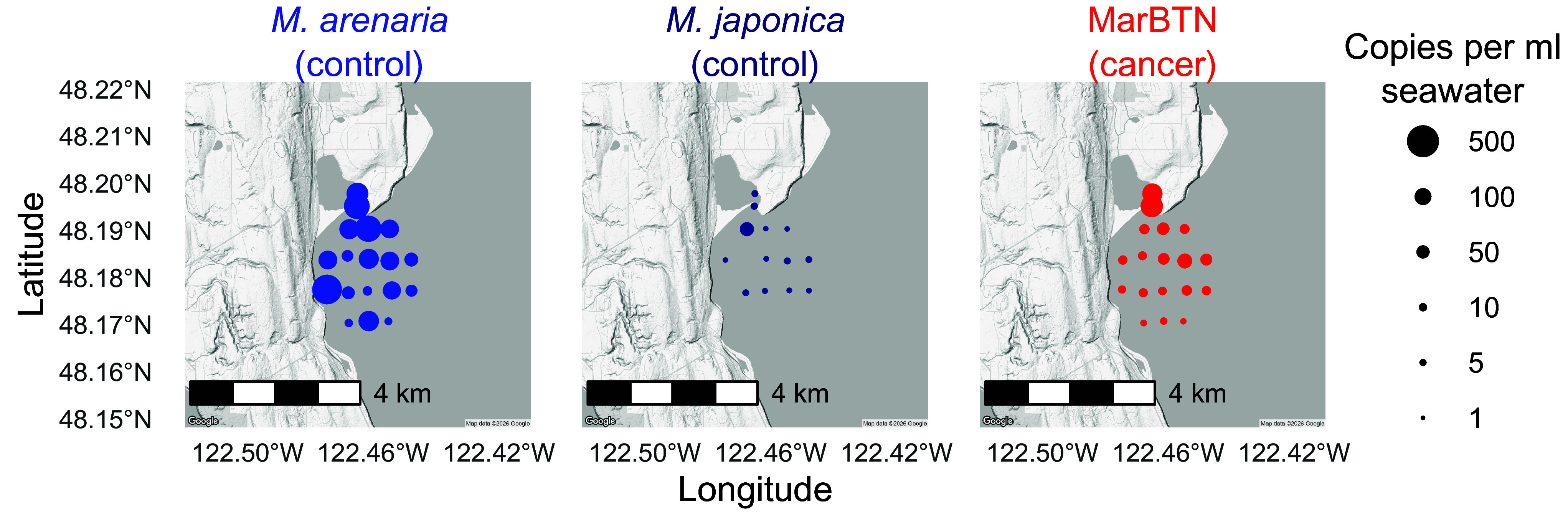
Detection of BTN in environmental DNA (eDNA) at a site of disease outbreak (Triangle Cove, Camano Island, WA). Samples of seawater were taken by boat at the mouth of Triangle Cove, Camano Island, Washington, and at multiple sites further from the entrance. The amplification from control primers (which amplify all *M. arenaria* mitochondrial DNA, *Left*, and *M. japonica* mitochondrial DNA, center) and from cancer-specific primers (which target two somatic mutations unique to the USA-sublineage of MarBTN, *Right*) are shown, with target quantity shown by the size of the point. Points are the average of two samples taken in a single day. Absolute quantification was calculated as copies of the target DNA per reaction using a standard plasmid control, normalized to copies per mL of seawater, assuming 100% efficiency of eDNA extraction from water samples. All clam collections in this study took place outside the cove itself, which was too shallow for sampling by boat.

Collection and diagnosis of clams is highly sensitive, but it requires considerable work at each collection that limits the widespread use of this method to track the spread of transmissible cancer across large areas. Previously, we used eDNA to detect MarBTN-specific DNA in aquarium water of diseased clams, but the sensitivity of the assay limited detection in wild samples (17). We therefore developed qPCR primers that targeted somatic SNVs in the mitochondrial genome of MarBTN. There are many copies of the mitochondrial genome per cell, so these targets can be much more sensitive than nuclear loci. These primers were first applied to seawater samples collected from around the entrance to Triangle Cove, WA, an area with known cancer spread. Water samples at the entrance to the cove were strongly positive for cancerous DNA, reaching 406 copies per mL, with lower levels of MarBTN detected in the seawater at greater distances from the mouth of the cove (Fig. 4). Interestingly, the *M. arenaria* control primers, which amplify the same mitochondrial locus targeting conserved sites, amplified to higher levels than the cancer primers at some of the sites furthest from the cove. This could be explained if MarBTN cells do not survive intact longer in seawater than the sources of normal clam eDNA, leading to more degradation of MarBTN eDNA, or if there are populations of clams with lower prevalence of the disease outside of the cove itself. These data show that transmissible cancer can be detected through eDNA sampling of seawater, and that, while the amount of MarBTN mtDNA decreases over distance from the cove, it remains detectable at 51 copies per mL as far as 2.8 km from the putative source of the cells.

### Sensitive Mitochondrial qPCR on eDNA Shows the Spread of MarBTN Throughout Puget Sound, Washington, the United States.

**Fig. 5.**
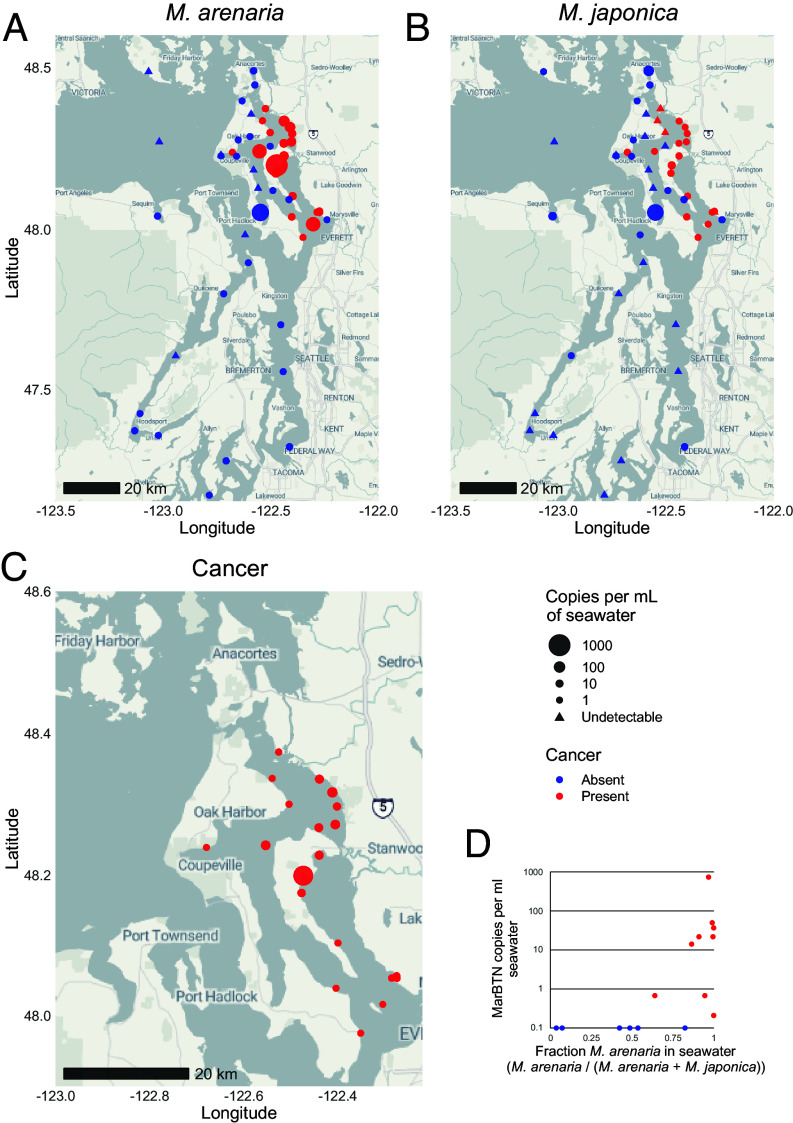
Spatial distribution of MarBTN outbreak shown by broad eDNA surveys across the Puget Sound. In 2023 and 2024, samples of water were collected from 50 locations in Puget Sound, Washington. Environmental DNA (eDNA) was extracted and samples were tested for the presence (circle) or absence (triangle) of DNA from soft-shell clams [*M. arenaria*, (*A*); *M. japonica*, (*B*)], using control primers. The points on the map show collection locations and the size of the point shows the quantity of the control target. The color denotes whether MarBTN-specific DNA was detected (red) or absent (blue). (*C*) A more detailed map of the area of the outbreak shows all points where MarBTN was detected, and the size of the points show the quantity of the MarBTN-specific amplification. Absolute quantification was calculated as copies of target DNA per reaction using a standard plasmid control, normalized to copies per mL of seawater, assuming 100% efficiency of eDNA extraction from water samples. Maps were generated using R packages ggplot (40), ggmap (41), and dplyr (42). Map style was generated by StadiaMaps, using map type “alidade_smooth.” Data in Table 2, *SI Appendix*, Table S3 (for sites at which multiple depth measurements were taken, the sample with the highest *M. arenaria* + *M. japonica* value was used for all maps). (*D*) The correlation between detection of MarBTN and species distribution is shown by calculating the fraction of each eDNA collection that is *M. arenaria*. The control *M. arenaria* primers amplify normal clam DNA and MarBTN, so to quantify only normal *M. arenaria* hosts, we subtract the MarBTN value from the *M. arenaria* control value, and then divide by the total amount of *M. arenaria* and *japonica* at each site. Sites with <10 copies per reaction of host clam DNA were excluded from this analysis. Linear regression of log10-transformed MarBTN values: y = 2.6691x − 1.7016; R = 0.4529.

To determine the spread of MarBTN throughout the Puget Sound, without the need for identifying specific clam populations or collecting and diagnosing individual animals, extensive eDNA sampling was performed across the region. After DNA extraction from collected seawater, samples were analyzed utilizing the qPCR assay developed to target mitochondrial somatic SNVs. Water samples were collected from shore at five sites in 2023, and by boat as well as from shore from 47 sites from 15 Mar through 27 Aug, 2024 (Fig. 5, Table 2, and *SI Appendix*, Table S3). Due to logistics of collecting eDNA by boat and by shore, slight variations in eDNA processing were used. To control for this, we used all methods on replicate samples taken from a spot with detectable MarBTN (*SI Appendix*, Fig. S4). Quantification of MarBTN from eDNA was highest when water was filtered immediately and filters were desiccated for storage before extraction with DNeasy PowerSoil Pro Kits (Qiagen). Alternative methods (freezing water prior to filtration or filter preservation in Longmire’s solution, followed by the modified CTAB protocol) were successful, but with lower yield. Overall, the results show overall variability of only about 2-fold between different methods.

**Table 2.** Sites in Puget Sound with positive detection of MarBTN in seawater using qPCR of eDNA

Site	Code	Latitude	Longitude	Date	MarBTN copies/mL of seawater	*M. arenaria* copies/mL of seawater	*M. japonica* copies/mL of seawater	*M. arenaria* *fraction* *
Cornet Bay	CB	48.397953	−122.634201	3/15/2024	0.14	0.27	0.11	0.54
Marthas Bay	MA	48.374039	−122.525592	3/15/2024	1.47	1.57	0.00	1.00
S Dugualla State Park	SD	48.336776	−122.539458	3/15/2024	0.65	0.89	0.00	1.00
Hall Slough	HS	48.335727	−122.43895	3/15/2024	22.37	77.51	0.30	0.99
Wiley Slough	WS	48.317002	−122.411458	3/15/2024	51.15	62.17	0.10	0.99
Strawberry Point	SP	48.300254	−122.50371	3/15/2024	1.11	1.95	0.00	1.00
Isohis Slough	IS	48.297092	−122.401538	3/15/2024	10.50	9.66	0.12	1.17
Oak Harbor	OK	48.277496	−122.651915	3/15/2024	0.12	0.18	0.11	0.39
Big Ditch Trail	BD	48.271592	−122.40523	3/15/2024	35.22	32.24	0.17	1.06
English Boom County Park	EB	48.267127	−122.440248	3/15/2024	20.00	18.73	0.13	1.11
Utsalady Bay	UD	48.258013	−122.50371	3/15/2024	0.17	0.25	0.00	1.00
Skagit Bay	P4-S	48.2422	−122.5533	7/8/2024	36.93	248.79	0.17	1.00
Penn Cove	PC	48.239139	−122.679333	12/11/2023	0.57	0.83	1.01	0.20
Livingston Bay	LB	48.22809	−122.439416	4/9/2024	22.24	32.27	1.01	0.91
Triangle Cove	TC	48.199121	−122.473384	8/27/2024	735.82	989.31	8.99	0.97
Cavalero Boat Launch	BL	48.174634	−122.477024	4/9/2024	13.96	21.94	1.27	0.86
Tillicum Beach	TL	48.103682	−122.399601	4/9/2024	0.67	10.80	0.60	0.94
Tulalip-2	TU-2	48.057059	−122.274826	3/29/2024	3.13	3.25	0.20	0.37
Tulalip-4	TU-4	48.053889	−122.285297	3/29/2024	2.02	2.91	0.25	0.79
Tulalip-3	TU-3	48.053545	−122.273967	3/29/2024	1.55	2.15	0.00	1.00
South Whidbey Harbor	SW	48.039403	−122.403646	4/15/2024	0.68	3.55	1.63	0.64
Gedney Island	P1-S	48.0165	−122.3042	7/8/2024	0.21	254.18	0.23	1.00
Clinton Ferry	CL	47.975502	−122.351307	4/15/2024	0.44	0.48	0.21	0.18

^*^Calculated using copies/mL seawater, subtracting MarBTN copies from total *M. arenaria* copies: (*M. arenaria* - MarBTN)/(*M. arenaria* - MarBTN + *M. japonica*)

Amplification with *M. arenaria* and *M. japonica*-specific primers identified soft-shell clam eDNA at most water collection sites, except for those farthest from shore and at three locations along the eastern coast of Whidbey Island, indicating regions where mitochondrial DNA from each of those species can be found (Fig. 5 *A* and *B*). The highest levels of *M. arenaria* and *M. japonica* amplification could be found at the known sites where we had previously collected clams, as well as some additional areas, such as southern Whidbey Island, around Everett, and further north in Skagit Bay, suggesting significant soft-shell clam populations in those areas. As expected, MarBTN was detected at high levels at Triangle Cove and Stanwood, reaching 735 copies per mL, but it was also observed at multiple sites further north into Skagit Bay, further south in Port Susan, and southwest Whidbey Island (Fig. 5). Notably, areas with detectable MarBTN coincide with those in which *M. arenaria* mtDNA dominates sample makeup, with a lower fraction of *M. japonica* mtDNA, and no MarBTN was detected in regions where *M. japonica* dominated sample makeup (Fig. 5*D*).

## Discussion

Through the analysis of clam hemolymph and a sensitive eDNA assay, we report the identification of an outbreak of MarBTN in soft-shell clams on the West Coast of the United States, in populations previously believed to be unexposed to the disease. Multiple genetic markers confirm that the MarBTN observed on the West Coast is most closely related to the sublineage that had previously only been found on the East Coast of the United States. It is possible that this cancer has been present in these populations undetected for some time; however, the lack of previous detection, rapidly increasing prevalence, and small geographic area affected all strongly suggest that this disease was recently transplanted from the East Coast to the West Coast. The long-distance transfer of MarBTN is likely due to an accidental human activity—transplantation of an infected clam or accidental transfer of seawater containing cancerous cells—but we have no evidence that would allow us to determine the exact mechanism of this transfer. Environmental variables are likely to affect susceptibility, and it is possible that the 2021 heat wave in the area could have increased the spread of this cancer. There are no records of intentional transfers of soft-shell clams from other states into Washington State in the last 50 y. There may have been some experimental introductions in the 1950–1960s, but those are likely the only intentional transfers since the introduction of soft-shell clams into the area in the 1800s (27, 28) (Washington Department of Fish and Wildlife). This would be the second case of a BTN spread to new oceans by human activities, after the MtrBTN2 lineage that has been observed in mussels in the *Mytilus* genus worldwide (8, 11). It is notable that mussels are known to adhere to ships and foul ballast water, making accidental long-distance transplantation more likely, but soft-shell clams do not spread in this way, so the exact route that brought MarBTN cells to Puget Sound, initiating this outbreak, remains unknown.

While the West Coast MarBTN is nearly identical to the East Coast sublineage, all West Coast samples are missing one *Steamer* retrotransposon insertion site. With the data available now it cannot be determined whether these West Coast cells split from the main East Coast USA sublineage prior to fixation of that site or whether that site was lost in the West Coast MarBTN. One common insertion site that was present in all other samples was absent in a single PEI sample (PEI-DN08), showing that deletion of an insertion site is possible. Similarly, the samples of MarBTN from Savage Harbour, PEI, may represent a previously uncharacterized sublineage of the cancer that diverged around the time the initial USA and PEI sublineages diverged, or even prior to that time. Also, while we have collected MarBTN samples from multiple locations on the East Coast, we have most likely not sampled the entirety of the MarBTN diversity. Further whole genome sequencing of these samples and of other samples across the East Coast will be needed to unravel the phylogenetic relationships of these sublineages. Regardless, these results demonstrate that there is more genomic diversity in circulating transmissible cancer cells than previously known.

There have been previous significant outbreaks of DN on the East Coast of the United States, of up to 90% prevalence (23, 24), that correlated with severe population die-offs and are likely to have been caused by MarBTN (although they occurred before the transmissible etiology of the disease was known). However, this disease is currently maintained in clam populations on the East Coast at low levels throughout all known populations in the region and has not led to any recent severe population losses. Moreover, based on genomic analysis of MarBTN, this lineage was estimated to be over 200 y old (26), showing that it has been coevolving with Eastern soft-shell clams for many generations. This outbreak of MarBTN in West Coast clams would be expected to follow a similar pattern in the future as the outbreak progresses, with significant mortality and severe population loss, followed by selection for more resistant clams, and finally enzootic maintenance of the disease in the population. While we have not quantitatively measured population changes over time, this outbreak correlates with anecdotal observations of population loss. Notably, a commercial clam-digging operation in south Skagit Bay that had been active for more than ten years had to stop collections in late 2024, due to dramatic mortality and population decreases in the area. The potential for local populations to face extirpation from this disease remains unclear, as does the timeline or nature of any potential evolutionary resistance.

Our results demonstrate hybridization between *M. arenaria* and *M. japonica* in the middle of this MarBTN outbreak. It is unclear if the clams sampled here with both *M. arenaria* and *M. japonica* sequences are true hybrids or due to past or recent introgression between the species, but in either case it is clear that hybridization has occurred. BTN has not been previously reported in *M. japonica* either in American or Asian populations.

Importantly, these observations suggest that *M. japonica* may be less susceptible to MarBTN than *M. arenaria*. We observed a trend of lower MarBTN prevalence in clams with *M. japonica* DNA, but the numbers are low and the difference is not statistically significant (Fig. 3*D*). Additionally, we have only analyzed one nuclear locus and one mitochondrial locus, so the genotypes here likely do not fully represent host genotypes, and they are an imperfect proxy for *M. japonica* ancestry. Additionally, using eDNA we observed an association between detection of MarBTN in seawater and locations that have more *M. arenaria* than *M. japonica* eDNA (Fig. 5*D*), however, this is only a single localized outbreak, so this pattern could also be caused by a founder effect. If it is true that *M. japonica* are less susceptible to MarBTN, then it seems likely that hybridization will lead to introgression of cancer-protective alleles in the affected populations. MarBTN has been shown to be lethal (25), and it should lead to a selective sweep of protective alleles in the mixed populations of Triangle Cove and other areas. As we only genotyped clams at one nuclear locus and one mitochondrial locus, the true extent of hybridization is unknown, and it is possible that some loci have already introgressed from one species into the other in these populations. Further genomic analysis of these populations will be needed to determine the extent of hybridization of these species and to determine whether there are *M. japonica* alleles that are able to protect clams from infection or mortality due to this transmissible cancer.

This study uses eDNA sampling to detect transmissible cancer in wild seawater samples in addition to mapping populations of closely related clams. The data show geographic trends in the relative abundance of *M. arenaria* and *M. japonica* mtDNA, revealing regions in which the mtDNA from one species dominates, and other regions with more even detection of the two species. This likely indicates large-scale differences in soft-shell clam population makeup across the Puget Sound, which is further supported by the genotyping of individuals collected across the region. Despite this, the eDNA analysis is based solely on mitochondrial genotype, so inferred trends in population makeup may be skewed in highly hybridized populations. Additionally, as MarBTN is itself *M. arenaria*, areas with high levels of cancer spread show inflated levels of *M. arenaria* signal, and the quantity of MarBTN mtDNA must be subtracted in order to determine the fraction of the signal that is due to *M. arenaria* clams alone.

These eDNA results reveal that the outbreak has spread beyond the initial sites that we have identified through collection and diagnosis of individual clams, but they also show that only a small subset of the soft-shell population in the Puget Sound is currently affected, with several large populations not yet impacted by MarBTN seemingly present on either side of the current outbreak. The rate at which BTNs spread from one population to another along the coast is unknown, but could feasibly be investigated through eDNA analysis and continued collection and diagnosis from sentinel populations.

BTNs have only recently been identified as infectious diseases, and this eDNA method will be vital for observing disease dynamics in the environment over time, and could be adapted to BTNs in other species as needed. This method could also be used in future analyses determining the association of MarBTN with variable environmental conditions once the disease has spread beyond a single focal outbreak, as has been previously done for other BTNs (49). This study shows the utility of eDNA in tracking and monitoring transmissible cancers, and we find that immediate seawater sample filtration and filter desiccation followed by extraction with DNeasy PowerSoil Pro Kits (Qiagen) is the best-practice BTN eDNA monitoring protocol, based on the methods we tested. If lacking access to a vacuum manifold, water samples can be frozen until filtration with some loss to yield. Assuming access to a vacuum manifold, but no freezer, filters can be preserved in Longmire’s solution before CTAB extraction, although mtDNA yield will likely be significantly reduced.

The recognition of this outbreak at an early stage makes it a critical case for understanding the mechanisms and speed of spread of this type of infection in the environment. This outbreak also provides a unique opportunity to study evolution of resistance to cancer in hybridizing wild populations in real time.

## Supplementary Material

Appendix 01 (PDF)

## Data Availability

Sequence data have been deposited in GenBank (BioProject PRJNA1476288; accession numbers for mtCOI: PZ222001.1-PZ222017.1; and EF1α: PZ233404.1-PZ233407.1) (50). Study data are included in the article and/or *SI Appendix*.
