# Supplementary material for: Atlantic to Pacific: Outbreak of bivalve transmissible neoplasia detected in hybridizing soft-shell clams and eDNA in Puget Sound
Source: Proc Natl Acad Sci U S A. 2026 Jun 23;123(26):e2611852123. doi: 10.1073/pnas.2611852123 (PMC13320677; doi:10.1073/pnas.2611852123)
Supplement: Supplementary file 1 — Appendix 01 (PDF) [file pnas.2611852123.sapp.pdf]

SUPPORTING INFORMATION APPENDIX

From the manuscript

Atlantic to Pacific: Outbreak of bivalve transmissible neoplasia detected in hybridizing soft-shell clams and eDNA in Puget Sound

Sydney A. Weinandt[1,2]*, Zachary J. Child[1]*, Dorothy Lartey[1,3], Angel Santos[4,5], Holden Maxfield[4,6], Jordana K. Sevigny[1,7], Fiona E. S. Garrett[1], Peter D. Smith[1], Rachael M. Giersch[1,8], Samuel F. M. Hart[1,9], Lucas Rabins[10], Samuel Kaiser[10], Anna Boyar[11], Jan Newton[11], Jesse Kerr[12], Franchesca Perez[13], James L. Dimond[4], and Michael J. Metzger[1]

* these authors contributed equally

Corresponding author: Michael J Metzger (metzgerm@pnri.org)

SI APPENDIX TABLE OF CONTENTS

Figure S1. Comparison of *Mya arenaria* and *Mya japonica* morphology
Figure S2. Standard curves for nuclear and mitochondrial qPCR assays
Figure S3. Methods for genotyping soft-shell clams as *M. arenaria* or *M. japonica* at two loci
Figure S4. Comparison of eDNA extraction methods
Figure S5. Quantification of disease severity in soft-shell clams with MarBTN
Table S1. Soft-shell clam collection and diagnosis for MarBTN in multiple sites in Puget Sound, WA, USA
Table S2. Primers used
Table S3. Quantification of MarBTN in eDNA from seawater collected from multiple sites in Puget Sound, WA, USA
Text S1. Extended protocol: Environmental DNA extraction protocol using Qiagen DNeasy Power Soil Pro & full reagent list

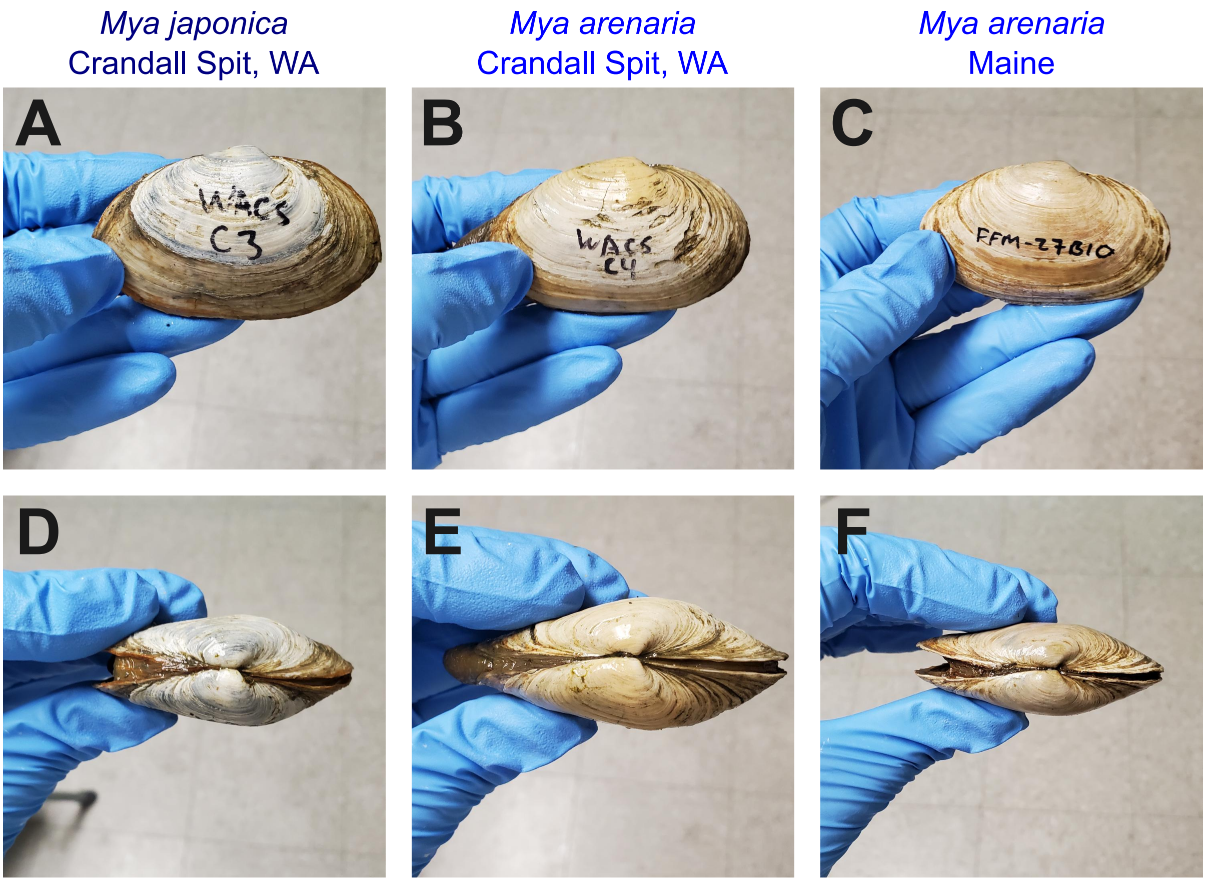

Figure S1. Comparison of *Mya arenaria* and *Mya japonica* morphology
The two soft-shell clam species in Puget Sound, WA, cannot be reliably distinguished based on morphology. Representative individuals shown here have been genotyped as (***A*,*D***) *Mya japonica* and (***B*,*E***) *Mya arenaria* at both a mitochondrial locus (*mtCOI*) and both copies of a nuclear locus (*EF1alpha*). Both of these were collected from Crandall Spit, WA. A separate representative of (***C*,*F***) *Mya arenaria* from Maine is also shown.

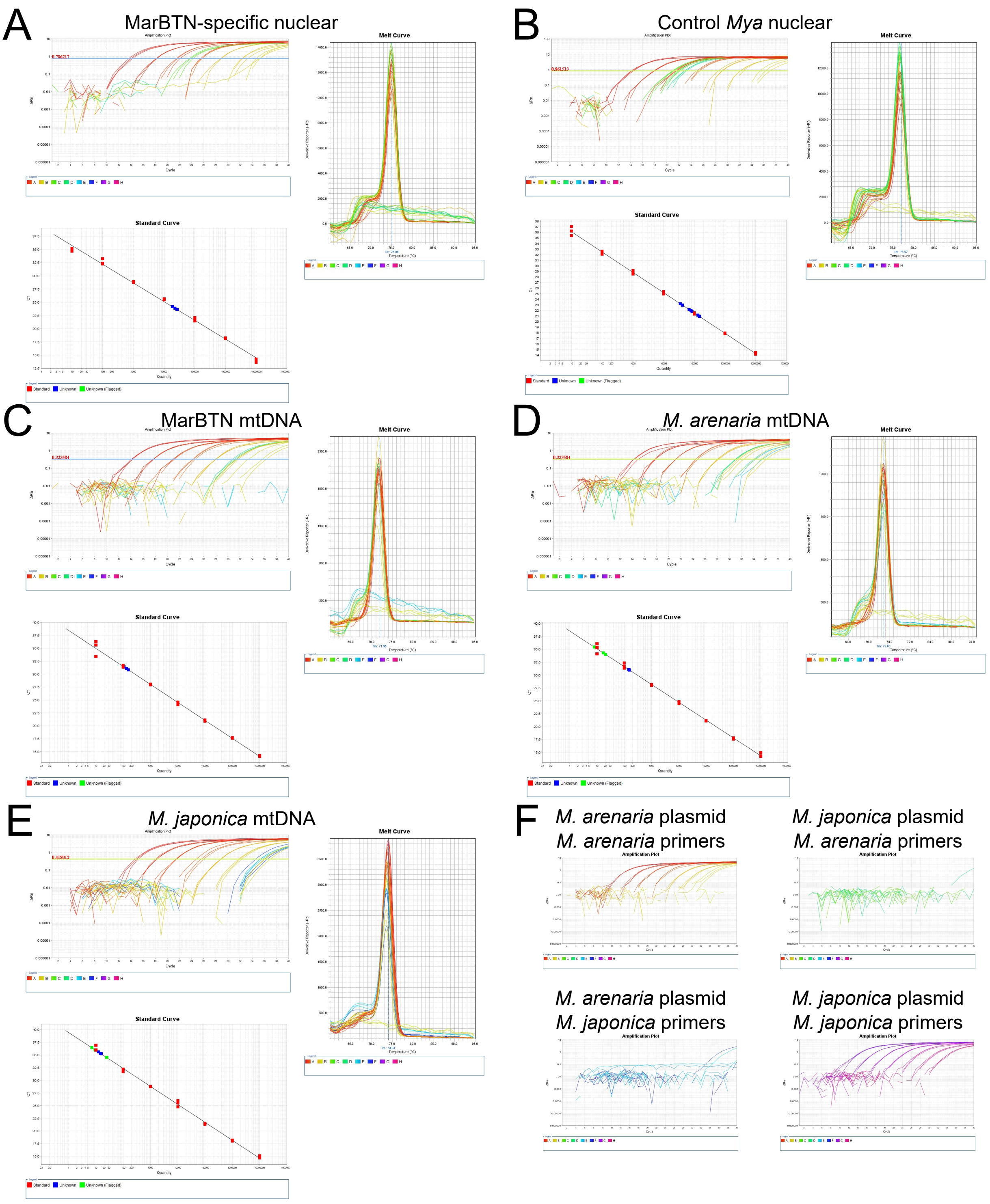

Figure S2. Standard curves for nuclear and mitochondrial qPCR assays

Diagnosis of MarBTN in soft-shell clams (*Mya arenaria* and *Mya japonica*) was done with a qPCR assay with two primer pairs: (***A***) one amplifies a Mar-BTN-specific insertion of a retrotransposon, and (***B***) the other conserved sites in the *N1N2* gene nearby. Both were described previously (17, 36). For each pair, the amplification plot, standard curve, and melt curve are shown for the plasmid standard curve (10^7-10^1 copies per reaction), a no-template control, and three samples (WATC-H3, 24% MarBTN positive; WATC-H4, MarBTN negative, *M. arenaria*; and WATC-H8, MarBTN negative, *M. japonica*). The samples are blue or green squares in the standard curve and green lines in other plots (showing amplification of only the positive sample with MarBTN primers, and amplification of all three *Mya* samples with the control primers. All samples are in triplicate. For MarBTN: slope = -3.543; R^2 = 0.995; efficiency = 91.538. For N1N2 control: slope = -3.638; R^2 = 0.998; efficiency = 87.315. For eDNA, qPCR primers target: (***C***) MarBTN-specific somatic mtDNA SNVs, (***D***) all *M. arenaria* sequence at the same mtDNA locus, (***E***) all *M. japonica* sequence at the same locus. Plots are shown as above, including three example eDNA samples (from Big Ditch Trail, BD-1, MarBTN positive; and Similk Bay SK-1, MarBTN negative). For MarBTN: slope = -3.482; R^2 = 0.995; efficiency = 93.727; for *M. arenaria* control: slope = -3.463; R^2 = 0.997; efficiency = 94.432; for *M. japonica* control: slope = -3.545; R^2 = 0.997; efficiency = 91.457. All primers listed in Table S2. (***F***) A direct test of species-specificity of mtDNA primers was done using full plasmid dilution series, showing no cross-species amplification (with the exception of sporatic and very late amplification in the wells with 10^7 copies of plasmid). Lines colors are based on position in the plate (rows A-H), and have no other meaning.

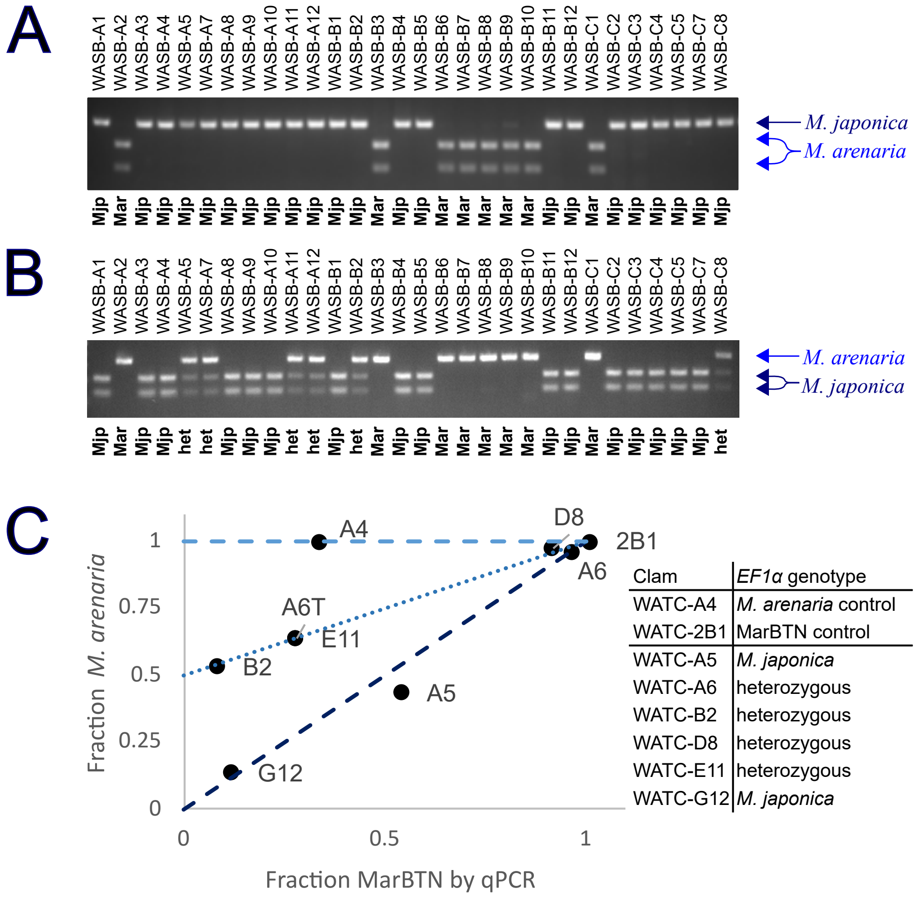

Figure S3. Methods for genotyping soft-shell clams as *M. arenaria* or *M. japonica* at two loci

Soft-shell clam populations in Puget Sound, Washington, USA, are a mixture of *M. arenaria* and *M. japonica*. We amplified two loci and used species-specific restriction digests to genotype all clams collected in the study. An example of a diagnostic gel is shown above for clams collected from Similk Bay. (***A***) For *mtCOI*, running the digested PCR product on a gel could distinguish amplicons that came from *M. arenaria* (255 and 457 bp bands) or *M. japonica* (intact 712 bp band). (***B***) For *EF1α*, the digest was able to distinguish homozygous *M. arenaria* (intact 334 bp band), homozygous *M. japonica* (120 and 214 bp bands), and clams heterozygous at that locus (120, 214, and 334 bp bands). Clam ID is listed above each lane and genotype is shown below (*M. arenaria,* Mar; *M. japonica,* Mjp; heterozygous, het). These data show the presence of clams heterozygous at the nuclear locus and clams with discordant nuclear and mitochondrial genotypes. (***C***) MarBTN is *M. arenaria*, so in clams with >10% MarBTN, we used a *M. japonica*-specific PCR to test for the presence of *M. japonica* DNA. For clams with BTN which showed PCR evidence of *M. japonica EF1α* sequence, we used sequencing (>5,000X coverage, Plasmidsaurus) to determine whether a clam was homozygous or heterozygous for that locus. The plot compares the fraction of cells in a sample that were MarBTN, based on qPCR, with the fraction of the *EF1α* sequences that were *M. arenaria*, based an average of eight species-specific SNPs, with lines showing the predicted positions for clams that are *M. arenaria* (dashed light blue on top), *M. japonica* (dashed dark blue below), or heterozygous (dotted) at the locus. The six MarBTN-positive clams with *M. japonica EF1α* sequence are shown, along with two controls clams that were homozygous for *M. arenaria* at this locus (one with moderate, and one with very high MarBTN). For one clam (WATC-A6), the level of MarBTN in the hemolymph was too high to determine the host genotype, so a tissue sample was also used for diagnosis (labeled A6T). See Table S1 for all genotypes.

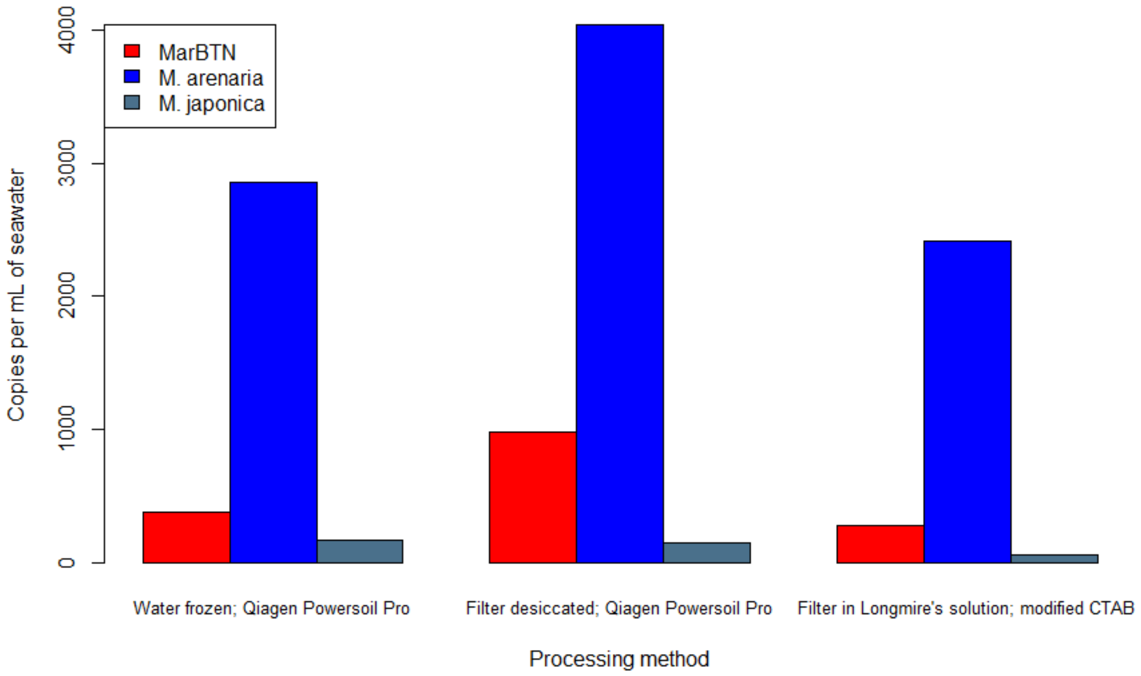

Figure S4. Comparison of eDNA extraction methods

In order to directly compare the eDNA yields from the slight variations in eDNA processing methods used in different collections, we collected three sets of triplicate water samples from a site in south Skagit Bay at which MarBTN had been detected (Big Ditch Trail, 48.271592, -122.40523). For each of the three processing and extraction methods, we extracted one set of triplicate water samples and compared the resulting qPCR values after amplification with all three primer sets (MarBTN, red; *M. arenaria*, blue; and *M. japonica*, gray). Higher yield were observed when water was filtered immediately after colelction and filters were desiccated for storage (center) than when water was frozen before filtration for storage before extraction with the Powersoil Pro kit (left), but both methods using the Powersoil Pro kit were showed higher yields than with the modified CTAB method (right). Overall, the results show overall variability of about 2-fold between different methods.

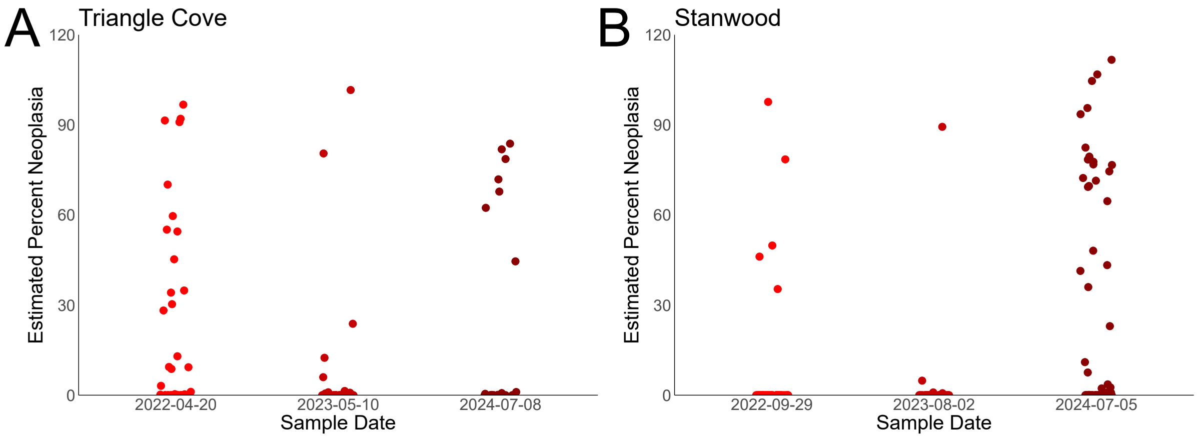

Figure S5. Quantification of disease severity in soft-shell clams with MarBTN
Soft-shell clams (*Mya arenaria* and *Mya japonica*) from two sites in Washington State were found to be positive for MarBTN based on collections from 2022-2024: (***A***) Triangle Cove, and (***B***) Stanwood (locations marked in map in Figure 1 and coordinates in Table 1). MarBTN diagnosis was made by qPCR analysis of hemolymph from collected clams, using a primer pair specific to a nuclear marker in MarBTN and a pair that amplifies that locus from all known soft-shell clam alleles. The estimated percent neoplasia in the hemolymph is shown, with a dot for each clam sampled. The estimated percent neoplasia for one clam collected from Triangle Cove in 2024 was higher than expected (WATC-2C10, 173%), which is not physiologically possible, and may reflect a deletion or mutation in the allele that does not contain the cancer-specific sequence (in most MarBTN samples from the USA sublineage, the target locus is tetraploid, with 4 alleles amplified by control primers and 2 alleles amplified by MarBTN-specific primers, and the calculation of percent neoplasia assumes this ploidy). This data point is not displayed on the figure, but it is listed in Table 1 as one of the eight clams from that collection with MarBTN >10%, and all values are listed in Table S1.

Table S1. Soft-shell clam collection and diagnosis for MarBTN in multiple sites in Puget Sound, WA, USA

Clam_ID	Location	Date received	N1N2 average	CANCER average	CANCER Wells_amp	MarBTN Fraction	Nuc Digest	Nuc Sanger	Nuc MjpPCR	Nuc DeepSeq	Nuc_Final Genotype	COI Digest	COI Sanger	COI MjpPCR	COI Genotype
WACS-A1	Crandall Spit	5/4/2022	67850.55	1	0	0	Japonica	Japonica			Japonica	Japonica	Japonica		Japonica
WACS-A10	Crandall Spit	5/4/2022	42365.33	1	0	0	Japonica	Japonica			Japonica	Japonica	Japonica		Japonica
WACS-A11	Crandall Spit	5/4/2022	78973.46	1	0	0	Japonica	Japonica			Japonica	Japonica	Japonica		Japonica
WACS-A12	Crandall Spit	5/4/2022	69159.24	1	0	0	Japonica	Japonica			Japonica	Japonica	Japonica		Japonica
WACS-A2	Crandall Spit	5/4/2022	76964.28	1	0	0	Japonica	Japonica			Japonica	Japonica	Japonica		Japonica
WACS-A3	Crandall Spit	5/4/2022	33448.43	1	0	0	Het	Het			Het	Japonica	Japonica		Japonica
WACS-A4	Crandall Spit	5/4/2022	36183.94	1	0	0	Arenaria	Arenaria			Arenaria	Arenaria	Arenaria		Arenaria
WACS-A5	Crandall Spit	5/4/2022	72048.54	1	0	0	Arenaria	Arenaria			Arenaria	Japonica	Japonica		Japonica
WACS-A6	Crandall Spit	5/4/2022	114715.2	1	0	0	Japonica	Japonica			Japonica	Japonica	Japonica		Japonica
WACS-A7	Crandall Spit	5/4/2022	138246.3	1	0	0	Arenaria	Arenaria			Arenaria	Arenaria	Arenaria		Arenaria
WACS-A8	Crandall Spit	5/4/2022	45697.41	1	0	0	Het	Het			Het	Japonica	Japonica		Japonica
WACS-A9	Crandall Spit	5/4/2022	125268	1	0	0	Japonica	Japonica			Japonica	Japonica	Japonica		Japonica
WACS-B1	Crandall Spit	5/4/2022	65432.79	1	0	0	Arenaria	Arenaria			Arenaria	Inconclusive	Arenaria	neg	Arenaria
WACS-B10	Crandall Spit	5/4/2022	67517.64	1	0	0	Japonica	Japonica			Japonica	Japonica	Japonica		Japonica
WACS-B11	Crandall Spit	5/4/2022	58829.95	1	0	0	Japonica	Japonica			Japonica	Japonica	Japonica		Japonica
WACS-B12	Crandall Spit	5/4/2022	192491.3	1	0	0	Arenaria	Arenaria			Arenaria	Arenaria	Arenaria		Arenaria
WACS-B2	Crandall Spit	5/4/2022	48227.58	1	0	0	Het	Het			Het	Japonica	Japonica		Japonica
WACS-B3	Crandall Spit	5/4/2022	36395.83	1	0	0	Japonica	Japonica			Japonica	Japonica	Japonica		Japonica
WACS-B4	Crandall Spit	5/4/2022	58904.83	1	0	0	Het	Het			Het	Arenaria	Arenaria		Arenaria
WACS-B5	Crandall Spit	5/4/2022	42074.89	1	0	0	Japonica	Japonica			Japonica	Japonica	Japonica		Japonica
WACS-B6	Crandall Spit	5/4/2022	47554.21	1	0	0	Japonica	Japonica			Japonica	Japonica	Japonica		Japonica
WACS-B7	Crandall Spit	5/4/2022	56292.84	1	0	0	Het	Het			Het	Japonica	Japonica		Japonica
WACS-B8	Crandall Spit	5/4/2022	65321.69	1	0	0	Japonica	Japonica			Japonica	Japonica	Japonica		Japonica
WACS-B9	Crandall Spit	5/4/2022	21356.1	1	0	0	Het	Het			Het	Japonica	Japonica		Japonica
WACS-C1	Crandall Spit	5/4/2022	26001.17	1	0	0	Japonica	Japonica			Japonica	Japonica	Japonica		Japonica
WACS-C10	Crandall Spit	5/4/2022	20666.41	1	0	0	Japonica	Japonica			Japonica	Japonica	Japonica		Japonica
WACS-C11	Crandall Spit	5/4/2022	21220.48	1	0	0	Het	Het			Het	Japonica	Japonica		Japonica
WACS-C12	Crandall Spit	5/4/2022	55587.65	1	0	0	Japonica	Japonica			Japonica	Japonica	Japonica		Japonica
WACS-C3	Crandall Spit	5/4/2022	52755.33	1	0	0	Japonica	Japonica			Japonica	Japonica	Japonica		Japonica
WACS-C4	Crandall Spit	5/4/2022	21348.49	1	0	0	Arenaria	Arenaria			Arenaria	Arenaria	Arenaria		Arenaria
WACS-C5	Crandall Spit	5/4/2022	31731.77	1	0	0	Japonica	Japonica			Japonica	Japonica	Japonica		Japonica
WACS-C6	Crandall Spit	5/4/2022	7052.95	1	0	0	Japonica	Japonica			Japonica	Japonica	Japonica		Japonica
WACS-C7	Crandall Spit	5/4/2022	22109.34	1	0	0	Het	Het			Het	Japonica	Japonica		Japonica
WACS-C8	Crandall Spit	5/4/2022	35990.38	1	0	0	Japonica	Japonica			Japonica	Japonica	Japonica		Japonica
WACS-C9	Crandall Spit	5/4/2022	24782.7	1	0	0	Japonica	Japonica			Japonica	Japonica	Japonica		Japonica
WACS-D1	Crandall Spit	5/4/2022	17153.78	1	0	0	Japonica	Japonica			Japonica	Japonica	Japonica		Japonica
WACS-D10	Crandall Spit	5/4/2022	28017.28	1	0	0	Arenaria	Arenaria			Arenaria	Arenaria	Arenaria		Arenaria
WACS-D11	Crandall Spit	5/4/2022	46274.76	1	0	0	Japonica	Japonica			Japonica	Japonica	Japonica		Japonica
WACS-D12	Crandall Spit	5/4/2022	35230.19	1	0	0	Japonica	Japonica			Japonica	Japonica	Japonica		Japonica
WACS-D2	Crandall Spit	5/4/2022	28371.04	1	0	0	Het	Het			Het	Japonica	Japonica		Japonica
WACS-D3	Crandall Spit	5/4/2022	58245.79	1	0	0	Japonica	Japonica			Japonica	Japonica	Japonica		Japonica
WACS-D4	Crandall Spit	5/4/2022	26072.41	1	0	0	Japonica	Japonica			Japonica	Japonica	Japonica		Japonica
WACS-D5	Crandall Spit	5/4/2022	37774.45	1	0	0	Het	Het			Het	Japonica	Japonica		Japonica

**WACS-D6**	**Crandall Spit**	**5/4/2022**	41137.7	1	0	**0**	Japonica	Japonica	**Japonica**	Japonica	Japonica	**Japonica**
**WACS-D7**	**Crandall Spit**	**5/4/2022**	33420.18	1	0	**0**	Het	Het	**Het**	Japonica	Japonica	**Japonica**
**WACS-D8**	**Crandall Spit**	**5/4/2022**	39534.49	1	0	**0**	Japonica	Japonica	**Japonica**	Japonica	Japonica	**Japonica**
**WACS-D9**	**Crandall Spit**	**5/4/2022**	22044.34	1	0	**0**	Het	Het	**Het**	Japonica	Japonica	**Japonica**
**WACS-E1**	**Crandall Spit**	**5/4/2022**	36322.06	1	0	**0**	Japonica	Japonica	**Japonica**	Japonica	Japonica	**Japonica**
**WACS-E10**	**Crandall Spit**	**6/6/2023**	228316.9	1	0	**0**	Japonica		**Japonica**	Japonica		**Japonica**
**WACS-E11**	**Crandall Spit**	**6/6/2023**	180384.5	1	0	**0**	Het		**Het**	Japonica		**Japonica**
**WACS-E12**	**Crandall Spit**	**6/6/2023**	354790.1	1	0	**0**	Japonica		**Japonica**	Japonica		**Japonica**
**WACS-E2**	**Crandall Spit**	**6/6/2023**	401917.5	1	0	**0**	Japonica		**Japonica**	Japonica		**Japonica**
**WACS-E3**	**Crandall Spit**	**6/6/2023**	161239.2	1	0	**0**	Japonica		**Japonica**	Japonica		**Japonica**
**WACS-E4**	**Crandall Spit**	**6/6/2023**	152154.1	1	0	**0**	Japonica		**Japonica**	Japonica		**Japonica**
**WACS-E5**	**Crandall Spit**	**6/6/2023**	147802.5	1	0	**0**	Japonica		**Japonica**	Japonica		**Japonica**
**WACS-E6**	**Crandall Spit**	**6/6/2023**	31510.61	1	0	**0**	Het		**Het**	Japonica		**Japonica**
**WACS-E7**	**Crandall Spit**	**6/6/2023**	214905.7	1	0	**0**	Japonica		**Japonica**	Japonica		**Japonica**
**WACS-E8**	**Crandall Spit**	**6/6/2023**	455590.4	1	0	**0**	Arenaria		**Arenaria**	Arenaria		**Arenaria**
**WACS-E9**	**Crandall Spit**	**6/6/2023**	163474.4	1	0	**0**	Japonica		**Japonica**	Japonica		**Japonica**
**WACS-F10**	**Crandall Spit**	**6/6/2023**	193125.7	1	0	**0**	Japonica		**Japonica**	Japonica		**Japonica**
**WACS-F11**	**Crandall Spit**	**6/6/2023**	226761.2	1	0	**0**	Japonica		**Japonica**	Japonica		**Japonica**
**WACS-F12**	**Crandall Spit**	**6/6/2023**	323145.2	1	0	**0**	Japonica		**Japonica**	Japonica		**Japonica**
**WACS-F2**	**Crandall Spit**	**6/6/2023**	321850.3	1	0	**0**	Japonica		**Japonica**	Japonica		**Japonica**
**WACS-F3**	**Crandall Spit**	**6/6/2023**	120206.9	1	0	**0**	Japonica		**Japonica**	Japonica		**Japonica**
**WACS-F4**	**Crandall Spit**	**6/6/2023**	93891.61	1	0	**0**	Het		**Het**	Japonica		**Japonica**
**WACS-F5**	**Crandall Spit**	**6/6/2023**	207429.8	1	0	**0**	Japonica		**Japonica**	Japonica		**Japonica**
**WACS-F6**	**Crandall Spit**	**6/6/2023**	78432.05	1	0	**0**	Japonica		**Japonica**	Japonica		**Japonica**
**WACS-F7**	**Crandall Spit**	**6/6/2023**	161124.4	1	0	**0**	Japonica		**Japonica**	Japonica		**Japonica**
**WACS-F8**	**Crandall Spit**	**6/6/2023**	151899.3	1	0	**0**	Japonica		**Japonica**	Japonica		**Japonica**
**WACS-F9**	**Crandall Spit**	**6/6/2023**	88906.5	1	0	**0**	Japonica		**Japonica**	Japonica		**Japonica**
**WACS-G1**	**Crandall Spit**	**6/6/2023**	372607.7	1	0	**0**	Het		**Het**	Japonica		**Japonica**
**WACS-G10**	**Crandall Spit**	**6/6/2023**	70799.74	1	0	**0**	Japonica		**Japonica**	Japonica		**Japonica**
**WACS-G11**	**Crandall Spit**	**6/6/2023**	191198.1	1	0	**0**	Japonica		**Japonica**	Japonica		**Japonica**
**WACS-G12**	**Crandall Spit**	**6/6/2023**	20457.95	1	0	**0**	Het		**Het**	Japonica		**Japonica**
**WACS-G2**	**Crandall Spit**	**6/6/2023**	466013.5	1	0	**0**	Arenaria		**Arenaria**	Japonica		**Japonica**
**WACS-G3**	**Crandall Spit**	**6/6/2023**	80658.38	1	0	**0**	Japonica		**Japonica**	Japonica		**Japonica**
**WACS-G4**	**Crandall Spit**	**6/6/2023**	221544.6	1	0	**0**	Japonica		**Japonica**	Japonica		**Japonica**
**WACS-G5**	**Crandall Spit**	**6/6/2023**	185520.5	1	0	**0**	Het		**Het**	Japonica		**Japonica**
**WACS-G6**	**Crandall Spit**	**6/6/2023**	141037.4	1	0	**0**	Japonica		**Japonica**	Japonica		**Japonica**
**WACS-G7**	**Crandall Spit**	**6/6/2023**	155340.1	1	0	**0**	Het		**Het**	Arenaria		**Arenaria**
**WACS-G8**	**Crandall Spit**	**6/6/2023**	66628.96	1	0	**0**	Japonica		**Japonica**	Japonica		**Japonica**
**WACS-G9**	**Crandall Spit**	**6/6/2023**	154122.9	1	0	**0**	Het		**Het**	Japonica		**Japonica**
**WACS-2A1**	**Crandall Spit**	**6/20/2024**	2970.7	1	0	**0**	Japonica		**Japonica**	Japonica		**Japonica**
**WACS-2A10**	**Crandall Spit**	**6/20/2024**	55590.69	1	0	**0**	Japonica		**Japonica**	Japonica		**Japonica**
**WACS-2A11**	**Crandall Spit**	**6/20/2024**	20045.95	1	0	**0**	Japonica		**Japonica**	Japonica		**Japonica**
**WACS-2A12**	**Crandall Spit**	**6/20/2024**	3382.934	1	0	**0**	Japonica		**Japonica**	Japonica		**Japonica**
**WACS-2A2**	**Crandall Spit**	**6/20/2024**	18918.38	1	0	**0**	Japonica		**Japonica**	Japonica		**Japonica**
**WACS-2A3**	**Crandall Spit**	**6/20/2024**	3485.569	1	0	**0**	Het		**Het**	Japonica		**Japonica**
**WACS-2A4**	**Crandall Spit**	**6/20/2024**	6567.973	1	0	**0**	Het		**Het**	Japonica		**Japonica**

**WACS-2A5**	**Crandall Spit**	**6/20/2024**	8576.161	1	0	**0**	Het	**Het**	Japonica	**Japonica**
**WACS-2A6**	**Crandall Spit**	**6/20/2024**	41083.87	1	0	**0**	Japonica	**Japonica**	Japonica	**Japonica**
**WACS-2A7**	**Crandall Spit**	**6/20/2024**	54209.74	1	0	**0**	Japonica	**Japonica**	Japonica	**Japonica**
**WACS-2A8**	**Crandall Spit**	**6/20/2024**	81212.5	1	0	**0**	Japonica	**Japonica**	Japonica	**Japonica**
**WACS-2A9**	**Crandall Spit**	**6/20/2024**	11388.58	1	0	**0**	Het	**Het**	Japonica	**Japonica**
**WACS-2B1**	**Crandall Spit**	**6/20/2024**	68872.47	1	0	**0**	Japonica	**Japonica**	Japonica	**Japonica**
**WACS-2B2**	**Crandall Spit**	**6/20/2024**	39043.69	1	0	**0**	Japonica	**Japonica**	Japonica	**Japonica**
**WACS-2B3**	**Crandall Spit**	**6/20/2024**	28808.78	1	0	**0**	Japonica	**Japonica**	Japonica	**Japonica**
**WACS-H1**	**Crandall Spit**	**6/20/2024**	17087.06	1	0	**0**	Het	**Het**	Japonica	**Japonica**
**WACS-H10**	**Crandall Spit**	**6/20/2024**	29291.33	1	0	**0**	Japonica	**Japonica**	Japonica	**Japonica**
**WACS-H11**	**Crandall Spit**	**6/20/2024**	20644.11	1	0	**0**	Japonica	**Japonica**	Japonica	**Japonica**
**WACS-H12**	**Crandall Spit**	**6/20/2024**	89024.47	1	0	**0**	Japonica	**Japonica**	Japonica	**Japonica**
**WACS-H2**	**Crandall Spit**	**6/20/2024**	67900.74	1	0	**0**	Het	**Het**	Japonica	**Japonica**
**WACS-H3**	**Crandall Spit**	**6/20/2024**	25955.65	1	0	**0**	Japonica	**Japonica**	Japonica	**Japonica**
**WACS-H4**	**Crandall Spit**	**6/20/2024**	8611.214	1	0	**0**	Japonica	**Japonica**	Japonica	**Japonica**
**WACS-H5**	**Crandall Spit**	**6/20/2024**	56541.61	1	0	**0**	Japonica	**Japonica**	Japonica	**Japonica**
**WACS-H6**	**Crandall Spit**	**6/20/2024**	80777.59	1	0	**0**	Japonica	**Japonica**	Japonica	**Japonica**
**WACS-H7**	**Crandall Spit**	**6/20/2024**	36368.42	1	0	**0**	Japonica	**Japonica**	Japonica	**Japonica**
**WACS-H8**	**Crandall Spit**	**6/20/2024**	42717.02	1	0	**0**	Het	**Het**	Japonica	**Japonica**
**WACS-H9**	**Crandall Spit**	**6/20/2024**	57728.55	1	0	**0**	Japonica	**Japonica**	Japonica	**Japonica**
**WASQ-A2**	**Sequim Bay**	**8/30/2023**	250156.9	1	0	**0**	Japonica	**Japonica**	Japonica	**Japonica**
**WASQ-A3**	**Sequim Bay**	**8/30/2023**	68503.56	1	0	**0**	Japonica	**Japonica**	Japonica	**Japonica**
**WASQ-A4**	**Sequim Bay**	**8/30/2023**	15317.42	1	0	**0**	Japonica	**Japonica**	Japonica	**Japonica**
**WASQ-A12**	**Sequim Bay**	**2/7/2024**	20703.59	1	0	**0**	Japonica	**Japonica**	Japonica	**Japonica**
**WASQ-B1**	**Sequim Bay**	**2/7/2024**	50044.97	1	0	**0**	Japonica	**Japonica**	Japonica	**Japonica**
**WASQ-B10**	**Sequim Bay**	**2/7/2024**	103748.7	1	0	**0**	Japonica	**Japonica**	Japonica	**Japonica**
**WASQ-B11**	**Sequim Bay**	**2/7/2024**	26194.73	1	0	**0**	Japonica	**Japonica**	Japonica	**Japonica**
**WASQ-B12**	**Sequim Bay**	**2/7/2024**	2924.059	1	0	**0**	Japonica	**Japonica**	Japonica	**Japonica**
**WASQ-B2**	**Sequim Bay**	**2/7/2024**	34320.8	1	0	**0**	Japonica	**Japonica**	Japonica	**Japonica**
**WASQ-B3**	**Sequim Bay**	**2/7/2024**	71045.55	1	0	**0**	Japonica	**Japonica**	Japonica	**Japonica**
**WASQ-B4**	**Sequim Bay**	**2/7/2024**	16543.28	1	0	**0**	Japonica	**Japonica**	Japonica	**Japonica**
**WASQ-B5**	**Sequim Bay**	**2/7/2024**	59238.27	1	0	**0**	Japonica	**Japonica**	Japonica	**Japonica**
**WASQ-B6**	**Sequim Bay**	**2/7/2024**	53708.39	1	0	**0**	Japonica	**Japonica**	Japonica	**Japonica**
**WASQ-B7**	**Sequim Bay**	**2/7/2024**	96967.61	1	0	**0**	Japonica	**Japonica**	Japonica	**Japonica**
**WASQ-B8**	**Sequim Bay**	**2/7/2024**	33914.66	1	1	**0**	Het	**Het**	Japonica	**Japonica**
**WASQ-C1**	**Sequim Bay**	**2/7/2024**	3672.531	1	1	**0**	Japonica	**Japonica**	Japonica	**Japonica**
**WASB-A1**	**Similk Bay**	**7/6/2023**	123394.2	1	0	**0**	Japonica	**Japonica**	Japonica	**Japonica**
**WASB-A10**	**Similk Bay**	**7/6/2023**	89013.61	1	0	**0**	Japonica	**Japonica**	Japonica	**Japonica**
**WASB-A11**	**Similk Bay**	**7/6/2023**	272643.7	1	0	**0**	Het	**Het**	Japonica	**Japonica**
**WASB-A12**	**Similk Bay**	**7/6/2023**	319988.7	1	0	**0**	Het	**Het**	Japonica	**Japonica**
**WASB-A2**	**Similk Bay**	**7/6/2023**	109872.3	1	0	**0**	Arenaria	**Arenaria**	Arenaria	**Arenaria**
**WASB-A3**	**Similk Bay**	**7/6/2023**	71448.01	1	0	**0**	Japonica	**Japonica**	Japonica	**Japonica**
**WASB-A4**	**Similk Bay**	**7/6/2023**	65044.25	1	0	**0**	Japonica	**Japonica**	Japonica	**Japonica**
**WASB-A5**	**Similk Bay**	**7/6/2023**	63579.22	1	0	**0**	Het	**Het**	Japonica	**Japonica**
**WASB-A7**	**Similk Bay**	**7/6/2023**	210399.4	1	0	**0**	Het	**Het**	Japonica	**Japonica**
**WASB-A8**	**Similk Bay**	**7/6/2023**	80156.11	1	0	**0**	Japonica	**Japonica**	Japonica	**Japonica**

**WASB-A9**	**Similk Bay**	**7/6/2023**	71893.96	1	0	**0**	Japonica	**Japonica**	Japonica	**Japonica**
**WASB-B1**	**Similk Bay**	**7/6/2023**	195774.5	1	0	**0**	Japonica	**Japonica**	Japonica	**Japonica**
**WASB-B10**	**Similk Bay**	**7/6/2023**	122967.6	1	0	**0**	Arenaria	**Arenaria**	Arenaria	**Arenaria**
**WASB-B11**	**Similk Bay**	**7/6/2023**	207497	1	0	**0**	Japonica	**Japonica**	Japonica	**Japonica**
**WASB-B12**	**Similk Bay**	**7/6/2023**	188756.9	1	0	**0**	Japonica	**Japonica**	Japonica	**Japonica**
**WASB-B2**	**Similk Bay**	**7/6/2023**	199893.7	1	0	**0**	Het	**Het**	Japonica	**Japonica**
**WASB-B3**	**Similk Bay**	**7/6/2023**	264404.3	1	0	**0**	Arenaria	**Arenaria**	Arenaria	**Arenaria**
**WASB-B4**	**Similk Bay**	**7/6/2023**	276870.8	1	0	**0**	Japonica	**Japonica**	Japonica	**Japonica**
**WASB-B5**	**Similk Bay**	**7/6/2023**	413896.3	1	0	**0**	Japonica	**Japonica**	Japonica	**Japonica**
**WASB-B6**	**Similk Bay**	**7/6/2023**	187004.7	1	0	**0**	Arenaria	**Arenaria**	Arenaria	**Arenaria**
**WASB-B7**	**Similk Bay**	**7/6/2023**	225138	1	0	**0**	Arenaria	**Arenaria**	Arenaria	**Arenaria**
**WASB-B8**	**Similk Bay**	**7/6/2023**	232613.4	1	0	**0**	Arenaria	**Arenaria**	Arenaria	**Arenaria**
**WASB-B9**	**Similk Bay**	**7/6/2023**	60974.38	1	0	**0**	Arenaria	**Arenaria**	Arenaria	**Arenaria**
**WASB-C1**	**Similk Bay**	**7/6/2023**	216339.3	1	0	**0**	Arenaria	**Arenaria**	Arenaria	**Arenaria**
**WASB-C10**	**Similk Bay**	**7/6/2023**	137455.3	1	0	**0**	Arenaria	**Arenaria**	Arenaria	**Arenaria**
**WASB-C11**	**Similk Bay**	**7/6/2023**	103824.8	1	0	**0**	Het	**Het**	Japonica	**Japonica**
**WASB-C12**	**Similk Bay**	**7/6/2023**	165345.1	1	0	**0**	Japonica	**Japonica**	Japonica	**Japonica**
**WASB-C2**	**Similk Bay**	**7/6/2023**	81143.49	1	0	**0**	Japonica	**Japonica**	Japonica	**Japonica**
**WASB-C3**	**Similk Bay**	**7/6/2023**	107077.7	1	0	**0**	Japonica	**Japonica**	Japonica	**Japonica**
**WASB-C4**	**Similk Bay**	**7/6/2023**	50861.05	1	0	**0**	Japonica	**Japonica**	Japonica	**Japonica**
**WASB-C5**	**Similk Bay**	**7/6/2023**	99529.26	1	0	**0**	Japonica	**Japonica**	Japonica	**Japonica**
**WASB-C7**	**Similk Bay**	**7/6/2023**	129682.9	1	0	**0**	Japonica	**Japonica**	Japonica	**Japonica**
**WASB-C8**	**Similk Bay**	**7/6/2023**	10183.42	1	0	**0**	Het	**Het**	Japonica	**Japonica**
**WASB-C9**	**Similk Bay**	**7/6/2023**	73028.87	1	0	**0**	Japonica	**Japonica**	Japonica	**Japonica**
**WASB-D1**	**Similk Bay**	**7/6/2023**	131237	1	0	**0**	Japonica	**Japonica**	Japonica	**Japonica**
**WASB-D2**	**Similk Bay**	**7/6/2023**	39835.18	1	0	**0**	Het	**Het**	Arenaria	**Arenaria**
**WASB-D3**	**Similk Bay**	**7/6/2023**	119261.6	1	0	**0**	Het	**Het**	Japonica	**Japonica**
**WASB-D10**	**Similk Bay**	**7/22/2024**	79354.45	1	0	**0**	Arenaria	**Arenaria**	Arenaria	**Arenaria**
**WASB-D11**	**Similk Bay**	**7/22/2024**	103159.3	1	0	**0**	Japonica	**Japonica**	Japonica	**Japonica**
**WASB-D12**	**Similk Bay**	**7/22/2024**	99479.87	1	0	**0**	Arenaria	**Arenaria**	Arenaria	**Arenaria**
**WASB-D4**	**Similk Bay**	**7/22/2024**	62831.49	1	0	**0**	Arenaria	**Arenaria**	Arenaria	**Arenaria**
**WASB-D5**	**Similk Bay**	**7/22/2024**	85690.09	1	0	**0**	Arenaria	**Arenaria**	Arenaria	**Arenaria**
**WASB-D6**	**Similk Bay**	**7/22/2024**	89900.88	1	0	**0**	Arenaria	**Arenaria**	Arenaria	**Arenaria**
**WASB-D7**	**Similk Bay**	**7/22/2024**	24459.03	1	0	**0**	Arenaria	**Arenaria**	Japonica	**Japonica**
**WASB-D8**	**Similk Bay**	**7/22/2024**	127916.4	1	0	**0**	Arenaria	**Arenaria**	Arenaria	**Arenaria**
**WASB-D9**	**Similk Bay**	**7/22/2024**	12662.5	1	0	**0**	Japonica	**Japonica**	Japonica	**Japonica**
**WASB-E1**	**Similk Bay**	**7/22/2024**	71933.56	1	0	**0**	Arenaria	**Arenaria**	Arenaria	**Arenaria**
**WASB-E2**	**Similk Bay**	**7/22/2024**	97640.25	1	0	**0**	Arenaria	**Arenaria**	Arenaria	**Arenaria**
**WASB-E3**	**Similk Bay**	**7/22/2024**	48083.45	1	0	**0**	Arenaria	**Arenaria**	Arenaria	**Arenaria**
**WASB-E4**	**Similk Bay**	**7/22/2024**	35214.5	1	0	**0**	Het	**Het**	Japonica	**Japonica**
**WASB-E5**	**Similk Bay**	**7/22/2024**	23238.06	1	0	**0**	Japonica	**Japonica**	Japonica	**Japonica**
**WASB-E6**	**Similk Bay**	**7/22/2024**	61706.02	1	0	**0**	Het	**Het**	Japonica	**Japonica**
**WATO-A1**	**Stanwood**	**9/29/2022**	16768.26	1	0	**0**	Arenaria	**Arenaria**	Arenaria	**Arenaria**
**WATO-A10**	**Stanwood**	**9/29/2022**	13521.21	1	0	**0**	Arenaria	**Arenaria**	Arenaria	**Arenaria**
**WATO-A11**	**Stanwood**	**9/29/2022**	10846.71	1	0	**0**	Arenaria	**Arenaria**	Arenaria	**Arenaria**
**WATO-A12**	**Stanwood**	**9/29/2022**	11031.54	1.74396	2	**0**	Arenaria	**Arenaria**	Arenaria	**Arenaria**

WATO-A2	Stanwood	9/29/2022	8468.154	1	0	0	Arenaria		Arenaria	Arenaria		Arenaria
WATO-A3	Stanwood	9/29/2022	24998.52	10995	3	0.78515	Arenaria	neg	Arenaria	Arenaria	neg	Arenaria
WATO-A4	Stanwood	9/29/2022	13021.8	1	0	0	Arenaria		Arenaria	Arenaria		Arenaria
WATO-A5	Stanwood	9/29/2022	6401.372	1	0	0	Arenaria		Arenaria	Arenaria		Arenaria
WATO-A6	Stanwood	9/29/2022	16134.92	1	1	0	Arenaria		Arenaria	Arenaria		Arenaria
WATO-A7	Stanwood	9/29/2022	9812.985	1	0	0	Arenaria		Arenaria	Arenaria		Arenaria
WATO-A8	Stanwood	9/29/2022	8988.327	1	0	0	Arenaria		Arenaria	Arenaria		Arenaria
WATO-A9	Stanwood	9/29/2022	15676.63	1	0	0	Arenaria		Arenaria	Arenaria		Arenaria
WATO-B1	Stanwood	9/29/2022	5874.645	1	0	0	Arenaria		Arenaria	Arenaria		Arenaria
WATO-B10	Stanwood	9/29/2022	19428.29	1	0	0	Arenaria		Arenaria	Arenaria		Arenaria
WATO-B11	Stanwood	9/29/2022	1495.583	1	0	0	Arenaria		Arenaria	Arenaria		Arenaria
WATO-B12	Stanwood	9/29/2022	11777.48	1	1	0	Arenaria		Arenaria	Arenaria		Arenaria
WATO-B2	Stanwood	9/29/2022	14024.94	1	0	0	Arenaria		Arenaria	Arenaria		Arenaria
WATO-B3	Stanwood	9/29/2022	4516.654	1179.21	3	0.35333	Arenaria	neg	Arenaria	Arenaria	neg	Arenaria
WATO-B4	Stanwood	9/29/2022	12104.81	1	0	0	Arenaria		Arenaria	Arenaria		Arenaria
WATO-B5	Stanwood	9/29/2022	6762.871	1	0	0	Arenaria		Arenaria	Arenaria		Arenaria
WATO-B6	Stanwood	9/29/2022	7076.317	1	0	0	Arenaria		Arenaria	Arenaria		Arenaria
WATO-B7	Stanwood	9/29/2022	16911.34	1	0	0	Arenaria		Arenaria	Arenaria		Arenaria
WATO-B8	Stanwood	9/29/2022	11069.85	1	0	0	Arenaria		Arenaria	Arenaria		Arenaria
WATO-B9	Stanwood	9/29/2022	11524.29	1	0	0	Arenaria		Arenaria	Arenaria		Arenaria
WATO-C1	Stanwood	9/29/2022	4551.953	1	0	0	Arenaria		Arenaria	Arenaria		Arenaria
WATO-C10	Stanwood	9/29/2022	15808.23	1	0	0	Arenaria		Arenaria	Arenaria		Arenaria
WATO-C11	Stanwood	9/29/2022	3703.249	1	0	0	Arenaria		Arenaria	Arenaria		Arenaria
WATO-C12	Stanwood	9/29/2022	6047.432	1	0	0	Arenaria		Arenaria	Arenaria		Arenaria
WATO-C2	Stanwood	9/29/2022	13235.86	1	1	0	Arenaria		Arenaria	Arenaria		Arenaria
WATO-C3	Stanwood	9/29/2022	15248.24	1	0	0	Arenaria		Arenaria	Arenaria		Arenaria
WATO-C4	Stanwood	9/29/2022	9167.181	1	0	0	Arenaria		Arenaria	Arenaria		Arenaria
WATO-C5	Stanwood	9/29/2022	2267.421	1	0	0	Arenaria		Arenaria	Arenaria		Arenaria
WATO-C6	Stanwood	9/29/2022	97350.93	32382	3	0.49842	Arenaria	neg	Arenaria	Arenaria	neg	Arenaria
WATO-C7	Stanwood	9/29/2022	4406.478	1	0	0	Arenaria		Arenaria	Arenaria		Arenaria
WATO-C8	Stanwood	9/29/2022	11095.6	1	0	0	Arenaria		Arenaria	Arenaria		Arenaria
WATO-C9	Stanwood	9/29/2022	5531.922	1	0	0	Arenaria		Arenaria	Arenaria		Arenaria
WATO-D1	Stanwood	9/29/2022	42723.67	21107.7	3	0.97648	Arenaria	neg	Arenaria	Arenaria	neg	Arenaria
WATO-D2	Stanwood	9/29/2022	10505.74	3316.48	3	0.46131	Arenaria	neg	Arenaria	Arenaria	neg	Arenaria
WATO-D3	Stanwood	9/29/2022	54323.31	1	0	0	Arenaria		Arenaria	Arenaria		Arenaria
WATO-D10	Stanwood	8/2/2023	69117.46	1	1	0	Arenaria		Arenaria	Arenaria		Arenaria
WATO-D11	Stanwood	8/2/2023	27652.77	2.1957	3	7.94E-05	Arenaria		Arenaria	Arenaria		Arenaria
WATO-D12	Stanwood	8/2/2023	53646.32	1	0	0	Arenaria		Arenaria	Arenaria		Arenaria
WATO-D4	Stanwood	8/2/2023	151745.7	1	0	0	Arenaria		Arenaria	Arenaria		Arenaria
WATO-D5	Stanwood	8/2/2023	95499.51	1	1	0	Arenaria		Arenaria	Arenaria		Arenaria
WATO-D6	Stanwood	8/2/2023	97108.53	21.211	3	2.18E-04	Arenaria		Arenaria	Arenaria		Arenaria
WATO-D7	Stanwood	8/2/2023	266223.8	1.6282	2	0	Arenaria		Arenaria	Arenaria		Arenaria
WATO-D8	Stanwood	8/2/2023	46201.82	1	0	0	Arenaria		Arenaria	Arenaria		Arenaria
WATO-D9	Stanwood	8/2/2023	30207.71	1	0	0	Arenaria		Arenaria	Arenaria		Arenaria
WATO-E1	Stanwood	8/2/2023	58023.35	2.38289	3	4.11E-05	Arenaria		Arenaria	Inconclusive	neg	Arenaria
WATO-E10	Stanwood	8/2/2023	61071.79	25.9616	3	4.25E-04	Arenaria		Arenaria	Arenaria		Arenaria

**WATO-E11**	**Stanwood**	**8/2/2023**	32386.98	5.06275	3	**1.56E-04**	Arenaria		**Arenaria**	Arenaria		**Arenaria**
**WATO-E12**	**Stanwood**	**8/2/2023**	118696.4	1	0	**0**	Arenaria		**Arenaria**	Arenaria		**Arenaria**
**WATO-E2**	**Stanwood**	**8/2/2023**	94973.27	44817.1	3	**0.89355**	Arenaria	neg	**Arenaria**	Arenaria	neg	**Arenaria**
**WATO-E3**	**Stanwood**	**8/2/2023**	38409.65	1.48758	2	**0**	Arenaria		**Arenaria**	Arenaria		**Arenaria**
**WATO-E4**	**Stanwood**	**8/2/2023**	74056.53	1	0	**0**	Arenaria		**Arenaria**	Arenaria		**Arenaria**
**WATO-E5**	**Stanwood**	**8/2/2023**	14615.14	1	0	**0**	Arenaria		**Arenaria**	Inconclusive	neg	**Arenaria**
**WATO-E6**	**Stanwood**	**8/2/2023**	58108.87	4.03183	2	**0**	Arenaria		**Arenaria**	Arenaria		**Arenaria**
**WATO-E7**	**Stanwood**	**8/2/2023**	46419.6	425.34	3	**0.00925**	Arenaria		**Arenaria**	Arenaria		**Arenaria**
**WATO-E8**	**Stanwood**	**8/2/2023**	10435.76	1	0	**0**	Arenaria		**Arenaria**	Arenaria		**Arenaria**
**WATO-E9**	**Stanwood**	**8/2/2023**	47541.98	7.11715	3	**1.50E-04**	Arenaria		**Arenaria**	Arenaria		**Arenaria**
**WATO-F1**	**Stanwood**	**8/2/2023**	35249.11	1	0	**0**	Arenaria		**Arenaria**	Arenaria		**Arenaria**
**WATO-F2**	**Stanwood**	**8/2/2023**	64692.96	1	0	**0**	Arenaria		**Arenaria**	Arenaria		**Arenaria**
**WATO-F3**	**Stanwood**	**8/2/2023**	131199.8	1.57669	1	**0**	Arenaria		**Arenaria**	Arenaria		**Arenaria**
**WATO-F4**	**Stanwood**	**8/2/2023**	67459.85	440.269	3	**0.00657**	Arenaria		**Arenaria**	Arenaria		**Arenaria**
**WATO-F5**	**Stanwood**	**8/2/2023**	71404.87	1	0	**0**	Arenaria		**Arenaria**	Arenaria		**Arenaria**
**WATO-F6**	**Stanwood**	**8/2/2023**	33417.25	3.30775	3	**9.90E-05**	Arenaria		**Arenaria**	Arenaria		**Arenaria**
**WATO-F7**	**Stanwood**	**8/2/2023**	20158.74	1	0	**0**	Arenaria		**Arenaria**	Arenaria		**Arenaria**
**WATO-F8**	**Stanwood**	**8/2/2023**	58938.37	2719.06	3	**0.04837**	Arenaria		**Arenaria**	Arenaria		**Arenaria**
**WATO-F9**	**Stanwood**	**8/2/2023**	89272.81	1	0	**0**	Arenaria		**Arenaria**	Arenaria		**Arenaria**
**WATO-2A1**	**Stanwood**	**7/5/2024**	65435.95	13.3335	3	**2.04E-04**	Arenaria		**Arenaria**	Arenaria		**Arenaria**
**WATO-2A10**	**Stanwood**	**7/5/2024**	539539.8	237206	3	**0.78459**	Arenaria	neg	**Arenaria**	Arenaria	neg	**Arenaria**
**WATO-2A11**	**Stanwood**	**7/5/2024**	146984.7	1234.67	3	**0.00847**	Arenaria		**Arenaria**	Arenaria		**Arenaria**
**WATO-2A12**	**Stanwood**	**7/5/2024**	402365.5	174588	3	**0.76649**	Arenaria	neg	**Arenaria**	Arenaria	neg	**Arenaria**
**WATO-2A2**	**Stanwood**	**7/5/2024**	18279.94	462.641	3	**0.02597**	Arenaria		**Arenaria**	Arenaria		**Arenaria**
**WATO-2A3**	**Stanwood**	**7/5/2024**	276819.3	116172	3	**0.72315**	Arenaria	neg	**Arenaria**	Arenaria	neg	**Arenaria**
**WATO-2A4**	**Stanwood**	**7/5/2024**	173448.2	32410.8	3	**0.2298**	Arenaria	neg	**Arenaria**	Arenaria	neg	**Arenaria**
**WATO-2A5**	**Stanwood**	**7/5/2024**	140781.9	4906.42	3	**0.03611**	Arenaria		**Arenaria**	Arenaria		**Arenaria**
**WATO-2A6**	**Stanwood**	**7/5/2024**	347802.1	157157	3	**0.82434**	Arenaria	neg	**Arenaria**	Arenaria	neg	**Arenaria**
**WATO-2A7**	**Stanwood**	**7/5/2024**	177875.5	878.147	3	**0.00496**	Arenaria		**Arenaria**	Arenaria		**Arenaria**
**WATO-2A8**	**Stanwood**	**7/5/2024**	340180.6	133510	3	**0.646**	Arenaria	neg	**Arenaria**	Arenaria	neg	**Arenaria**
**WATO-2A9**	**Stanwood**	**7/5/2024**	715100.6	316563	3	**0.79431**	Arenaria	neg	**Arenaria**	Arenaria	neg	**Arenaria**
**WATO-2B1**	**Stanwood**	**7/5/2024**	489077.3	236394	3	**0.93553**	Arenaria	neg	**Arenaria**	Arenaria	neg	**Arenaria**
**WATO-2B10**	**Stanwood**	**7/5/2024**	32226.46	715.491	3	**0.02271**	Arenaria		**Arenaria**	Arenaria		**Arenaria**
**WATO-2B11**	**Stanwood**	**7/5/2024**	18221.03	6.95129	3	**3.82E-04**	Arenaria		**Arenaria**	Arenaria		**Arenaria**
**WATO-2B12**	**Stanwood**	**7/5/2024**	25600.17	5.52042	3	**2.16E-04**	Arenaria		**Arenaria**	Arenaria		**Arenaria**
**WATO-2B2**	**Stanwood**	**7/5/2024**	340725	145492	3	**0.74522**	Arenaria	neg	**Arenaria**	Arenaria	neg	**Arenaria**
**WATO-2B3**	**Stanwood**	**7/5/2024**	177194.7	1.47039	2	**0**	Arenaria		**Arenaria**	Arenaria		**Arenaria**
**WATO-2B4**	**Stanwood**	**7/5/2024**	150530.3	2.29134	2	**0**	Arenaria		**Arenaria**	Arenaria		**Arenaria**
**WATO-2B5**	**Stanwood**	**7/5/2024**	158483	1	1	**0**	Arenaria		**Arenaria**	Arenaria		**Arenaria**
**WATO-2B6**	**Stanwood**	**7/5/2024**	135031.2	9490.81	3	**0.0756**	Arenaria		**Arenaria**	Arenaria		**Arenaria**
**WATO-2B7**	**Stanwood**	**7/5/2024**	57746.24	1.93071	3	**3.34E-05**	Arenaria		**Arenaria**	Arenaria		**Arenaria**
**WATO-2B8**	**Stanwood**	**7/5/2024**	41885.72	20.1862	3	**4.82E-04**	Arenaria		**Arenaria**	Arenaria		**Arenaria**
**WATO-2B9**	**Stanwood**	**7/5/2024**	29715.89	1.66195	3	**5.59E-05**	Arenaria		**Arenaria**	Arenaria		**Arenaria**
**WATO-2C1**	**Stanwood**	**7/5/2024**	33586.78	3.79862	2	**0**	Arenaria		**Arenaria**	Arenaria		**Arenaria**
**WATO-2C11**	**Stanwood**	**7/5/2024**	22655.14	1	0	**0**	Arenaria		**Arenaria**	Arenaria		**Arenaria**
**WATO-2C12**	**Stanwood**	**7/5/2024**	18464.34	12.4041	3	**6.72E-04**	Arenaria		**Arenaria**	Arenaria		**Arenaria**

**WATO-2C3**	**Stanwood**	**7/5/2024**	36585.96	15243.1	3	**0.7142**	Arenaria		neg		**Arenaria**	Arenaria		neg	**Arenaria**
**WATO-2C4**	**Stanwood**	**7/5/2024**	33743.78	33.3645	3	**9.90E-04**	Arenaria				**Arenaria**	Arenaria			**Arenaria**
**WATO-2C5**	**Stanwood**	**7/5/2024**	21063.73	4.62661	3	**2.20E-04**	Arenaria				**Arenaria**	Arenaria			**Arenaria**
**WATO-2C6**	**Stanwood**	**7/5/2024**	65246.91	19097.5	3	**0.41382**	Arenaria		neg		**Arenaria**	Arenaria		neg	**Arenaria**
**WATO-2C7**	**Stanwood**	**7/5/2024**	28489.29	325.536	3	**0.01156**	Arenaria				**Arenaria**	Arenaria			**Arenaria**
**WATO-2C8**	**Stanwood**	**7/5/2024**	19018.53	13.4465	3	**7.08E-04**	Arenaria				**Arenaria**	Arenaria			**Arenaria**
**WATO-2C9**	**Stanwood**	**7/5/2024**	3081.823	1	0	**0**	Arenaria				**Arenaria**	Arenaria			**Arenaria**
**WATO-2D1**	**Stanwood**	**7/5/2024**	190815.9	83443.4	3	**0.77714**	Arenaria		neg		**Arenaria**	Arenaria		neg	**Arenaria**
**WATO-2D2**	**Stanwood**	**7/5/2024**	35204.47	1	0	**0**	Arenaria				**Arenaria**	Arenaria			**Arenaria**
**WATO-2D3**	**Stanwood**	**7/5/2024**	27792.85	1	0	**0**	Arenaria				**Arenaria**	Arenaria			**Arenaria**
**WATO-2D4**	**Stanwood**	**7/5/2024**	19372.71	13.8744	3	**7.17E-04**	Arenaria				**Arenaria**	Arenaria			**Arenaria**
**WATO-2D5**	**Stanwood**	**7/5/2024**	21561.53	2.89494	3	**1.34E-04**	Arenaria				**Arenaria**	Arenaria			**Arenaria**
**WATO-F10**	**Stanwood**	**7/5/2024**	406614.9	1.80518	1	**0**	Arenaria				**Arenaria**	Arenaria			**Arenaria**
**WATO-F11**	**Stanwood**	**7/5/2024**	159555.5	1	0	**0**	Arenaria				**Arenaria**	Arenaria			**Arenaria**
**WATO-F12**	**Stanwood**	**7/5/2024**	130275.2	34478.1	3	**0.35991**	Arenaria		neg		**Arenaria**	Arenaria		neg	**Arenaria**
**WATO-G1**	**Stanwood**	**7/5/2024**	252199	1	0	**0**	Arenaria				**Arenaria**	Arenaria			**Arenaria**
**WATO-G10**	**Stanwood**	**7/5/2024**	113159.3	34191.7	3	**0.43298**	Arenaria		neg		**Arenaria**	Arenaria		neg	**Arenaria**
**WATO-G11**	**Stanwood**	**7/5/2024**	544000.6	236372	3	**0.76837**	Arenaria		neg		**Arenaria**	Arenaria		neg	**Arenaria**
**WATO-G12**	**Stanwood**	**7/5/2024**	166712.7	396.624	3	**0.00238**	Arenaria				**Arenaria**	Arenaria			**Arenaria**
**WATO-G2**	**Stanwood**	**7/5/2024**	883786.6	389704	3	**0.78874**	Arenaria		neg		**Arenaria**	Arenaria		neg	**Arenaria**
**WATO-G3**	**Stanwood**	**7/5/2024**	1017428	560525	3	**1.22679**	Arenaria		neg		**Arenaria**	Arenaria		neg	**Arenaria**
**WATO-G4**	**Stanwood**	**7/5/2024**	1256507	662886	3	**1.11668**	Arenaria		neg		**Arenaria**	Arenaria		neg	**Arenaria**
**WATO-G5**	**Stanwood**	**7/5/2024**	245938	1.60637	1	**0**	Arenaria				**Arenaria**	Arenaria			**Arenaria**
**WATO-G6**	**Stanwood**	**7/5/2024**	297419.8	4.33711	2	**0**	Arenaria				**Arenaria**	Arenaria			**Arenaria**
**WATO-G7**	**Stanwood**	**7/5/2024**	73007.41	4.22642	3	**5.79E-05**	Arenaria				**Arenaria**	Japonica			**Japonica**
**WATO-G8**	**Stanwood**	**7/5/2024**	697119.5	285464	3	**0.69345**	Arenaria		neg		**Arenaria**	Arenaria		neg	**Arenaria**
**WATO-G9**	**Stanwood**	**7/5/2024**	782896	404337	3	**1.0681**	Arenaria		neg		**Arenaria**	Arenaria		neg	**Arenaria**
**WATO-H1**	**Stanwood**	**7/5/2024**	1034603	587102	3	**1.31196**	Arenaria		neg		**Arenaria**	Arenaria		neg	**Arenaria**
**WATO-H10**	**Stanwood**	**7/5/2024**	302346.7	147782	3	**0.95611**	Arenaria		neg		**Arenaria**	Arenaria		neg	**Arenaria**
**WATO-H11**	**Stanwood**	**7/5/2024**	115255.3	11424.9	3	**0.11003**	Arenaria		neg		**Arenaria**	Arenaria		neg	**Arenaria**
**WATO-H12**	**Stanwood**	**7/5/2024**	10948.42	3.75722	3	**3.43E-04**	Arenaria				**Arenaria**	Arenaria			**Arenaria**
**WATO-H2**	**Stanwood**	**7/5/2024**	199591.7	145.873	3	**7.31E-04**	Arenaria				**Arenaria**	Arenaria			**Arenaria**
**WATO-H3**	**Stanwood**	**7/5/2024**	349091.5	5.59935	2	**0**	Arenaria				**Arenaria**	Arenaria			**Arenaria**
**WATO-H4**	**Stanwood**	**7/5/2024**	518937.1	265329	3	**1.04621**	Arenaria		neg		**Arenaria**	Arenaria		neg	**Arenaria**
**WATO-H5**	**Stanwood**	**7/5/2024**	591221	242717	3	**0.69646**	Arenaria		neg		**Arenaria**	Arenaria		neg	**Arenaria**
**WATO-H6**	**Stanwood**	**7/5/2024**	49411.65	7.6069	2	**0**	Arenaria				**Arenaria**	Arenaria			**Arenaria**
**WATO-H7**	**Stanwood**	**7/5/2024**	141097.2	13.3775	3	**9.48E-05**	Arenaria				**Arenaria**	Arenaria			**Arenaria**
**WATO-H8**	**Stanwood**	**7/5/2024**	163660.1	4.52647	3	**2.77E-05**	Arenaria				**Arenaria**	Arenaria			**Arenaria**
**WATO-H9**	**Stanwood**	**7/5/2024**	212743	69101.4	3	**0.48107**	Arenaria		neg		**Arenaria**	Arenaria		neg	**Arenaria**
**WATC-A1**	**Triangle Cove**	**4/20/2022**	866276.6	99364.8	3	**0.12956**	Arenaria	Arenaria	neg		**Arenaria**	Arenaria	Arenaria	neg	**Arenaria**
**WATC-A10**	**Triangle Cove**	**4/20/2022**	509394.9	1	0	**0**	Arenaria	Arenaria			**Arenaria**	Arenaria	Arenaria		**Arenaria**
**WATC-A11**	**Triangle Cove**	**4/20/2022**	514005.7	1	0	**0**	Arenaria	Arenaria			**Arenaria**	Arenaria	Arenaria		**Arenaria**
**WATC-A12**	**Triangle Cove**	**4/20/2022**	351036.9	1	0	**0**	Arenaria	Arenaria			**Arenaria**	Arenaria	Arenaria		**Arenaria**
**WATC-A2**	**Triangle Cove**	**4/20/2022**	36297.85	9383.51	3	**0.34864**	Arenaria	Arenaria	neg		**Arenaria**	Arenaria	Arenaria	neg	**Arenaria**
**WATC-A3**	**Triangle Cove**	**4/20/2022**	267280	1	0	**0**	Arenaria	Arenaria			**Arenaria**	Arenaria	Arenaria		**Arenaria**
**WATC-A4**	**Triangle Cove**	**4/20/2022**	316733.3	80631	3	**0.34151**	Arenaria	Arenaria	neg	Arenaria	**Arenaria**	Arenaria	Arenaria	neg	**Arenaria**

**WATC-A5**	**Triangle Cove**	**4/20/2022**	359065.6	126677	3	**0.54511**	Het	Het	pos	Japonica	**Japonica**	Arenaria	Arenaria	neg	**Arenaria**
**WATC-A6**	**Triangle Cove**	**4/20/2022**	313239.2	154021	3	**0.96736**	Arenaria	Arenaria	pos	Het	**Het**	Arenaria	Arenaria	neg	**Arenaria**
**WATC-A7**	**Triangle Cove**	**4/20/2022**	376809.8	1	0	**0**	Arenaria	Arenaria			**Arenaria**	Arenaria	Arenaria		**Arenaria**
**WATC-A8**	**Triangle Cove**	**4/20/2022**	206303.7	1	0	**0**	Arenaria	Arenaria			**Arenaria**	Arenaria	Arenaria		**Arenaria**
**WATC-A9**	**Triangle Cove**	**4/20/2022**	459627.4	219573	3	**0.91468**	Arenaria	Arenaria	neg		**Arenaria**	Arenaria	Arenaria	neg	**Arenaria**
**WATC-B1**	**Triangle Cove**	**4/20/2022**	431749.2	1	0	**0**	Het	Het			**Het**	Arenaria	Arenaria		**Arenaria**
**WATC-B10**	**Triangle Cove**	**4/20/2022**	421794.7	1	0	**0**	Arenaria	Arenaria			**Arenaria**	Arenaria	Arenaria		**Arenaria**
**WATC-B11**	**Triangle Cove**	**4/20/2022**	386630.7	8.46691	1	**0**	Arenaria	Arenaria			**Arenaria**	Arenaria	Arenaria		**Arenaria**
**WATC-B12**	**Triangle Cove**	**4/20/2022**	347757.4	1	0	**0**	Arenaria	Arenaria			**Arenaria**	Arenaria	Arenaria		**Arenaria**
**WATC-B2**	**Triangle Cove**	**4/20/2022**	454218.5	36531	3	**0.08746**	Het	Het	pos	Het	**Het**	Arenaria	Arenaria	neg	**Arenaria**
**WATC-B3**	**Triangle Cove**	**4/20/2022**	448964.2	38224.3	3	**0.09306**	Arenaria	Arenaria	neg		**Arenaria**	Arenaria	Arenaria	neg	**Arenaria**
**WATC-B4**	**Triangle Cove**	**4/20/2022**	396797.2	1	0	**0**	Arenaria	Arenaria			**Arenaria**	Arenaria	Arenaria		**Arenaria**
**WATC-B5**	**Triangle Cove**	**4/20/2022**	274390.3	1	0	**0**	Arenaria	Arenaria			**Arenaria**	Arenaria	Arenaria		**Arenaria**
**WATC-B6**	**Triangle Cove**	**4/20/2022**	333573.9	96.0632	3	**2.88E-04**	Arenaria	Arenaria			**Arenaria**	Arenaria	Arenaria		**Arenaria**
**WATC-B7**	**Triangle Cove**	**4/20/2022**	588426.3	219827	3	**0.59638**	Arenaria	Arenaria	neg		**Arenaria**	Arenaria	Arenaria	neg	**Arenaria**
**WATC-B8**	**Triangle Cove**	**4/20/2022**	364527.6	1	0	**0**	Arenaria	Arenaria			**Arenaria**	Arenaria	Arenaria		**Arenaria**
**WATC-B9**	**Triangle Cove**	**4/20/2022**	333786.2	9.02098	2	**0**	Het	Het			**Het**	Arenaria	Arenaria		**Arenaria**
**WATC-C1**	**Triangle Cove**	**4/20/2022**	406228.7	8.59469	1	**0**	Het	Het			**Het**	Japonica	Japonica		**Japonica**
**WATC-C10**	**Triangle Cove**	**4/20/2022**	23188.64	30.6082	3	**0.00132**	Arenaria	Arenaria			**Arenaria**	Inconclusive	Arenaria	neg	**Arenaria**
**WATC-C11**	**Triangle Cove**	**4/20/2022**	46952.07	1	1	**0**	Japonica	Japonica			**Japonica**	Japonica	Japonica		**Japonica**
**WATC-C12**	**Triangle Cove**	**4/20/2022**	41323.03	1	0	**0**	Arenaria	Arenaria			**Arenaria**	Arenaria	Arenaria		**Arenaria**
**WATC-C2**	**Triangle Cove**	**4/20/2022**	438374.5	1	0	**0**	Arenaria	Arenaria			**Arenaria**	Arenaria	Arenaria		**Arenaria**
**WATC-C3**	**Triangle Cove**	**4/20/2022**	543612.2	1	0	**0**	Arenaria	Arenaria			**Arenaria**	Arenaria	Arenaria		**Arenaria**
**WATC-C4**	**Triangle Cove**	**4/20/2022**	272267.2	129658	3	**0.90919**	Arenaria	Arenaria	neg		**Arenaria**	Arenaria	Arenaria	neg	**Arenaria**
**WATC-C5**	**Triangle Cove**	**4/20/2022**	325576	16.7444	1	**0**	Arenaria	Arenaria			**Arenaria**	Arenaria	Arenaria		**Arenaria**
**WATC-C6**	**Triangle Cove**	**4/20/2022**	213672.9	88067.7	3	**0.70115**	Arenaria	Arenaria	neg		**Arenaria**	Arenaria	Arenaria	neg	**Arenaria**
**WATC-C7**	**Triangle Cove**	**4/20/2022**	274986.2	465.244	3	**0.00169**	Het	Het			**Het**	Arenaria	Arenaria		**Arenaria**
**WATC-C8**	**Triangle Cove**	**4/20/2022**	59662.21	200.121	3	**0.00337**	Het	Het			**Het**	Arenaria	Arenaria		**Arenaria**
**WATC-C9**	**Triangle Cove**	**4/20/2022**	34429.48	1	0	**0**	Arenaria	Arenaria			**Arenaria**	Arenaria	Arenaria		**Arenaria**
**WATC-D1**	**Triangle Cove**	**4/20/2022**	59987.02	1	0	**0**	Arenaria	Arenaria			**Arenaria**	Arenaria	Arenaria		**Arenaria**
**WATC-D10**	**Triangle Cove**	**4/20/2022**	131418.2	144.013	3	**0.0011**	Arenaria	Arenaria			**Arenaria**	Inconclusive	Japonica	neg	**Arenaria**
**WATC-D11**	**Triangle Cove**	**4/20/2022**	77840.05	1	0	**0**	Het	Het			**Het**	Arenaria	Arenaria		**Arenaria**
**WATC-D12**	**Triangle Cove**	**4/20/2022**	131669.4	1	0	**0**	Arenaria	Arenaria			**Arenaria**	Arenaria	Arenaria		**Arenaria**
**WATC-D2**	**Triangle Cove**	**4/20/2022**	61802.13	686.125	3	**0.01123**	Arenaria	Arenaria			**Arenaria**	Arenaria	Arenaria		**Arenaria**
**WATC-D3**	**Triangle Cove**	**4/20/2022**	56316.97	1	0	**0**	Arenaria	Arenaria			**Arenaria**	Inconclusive	Arenaria	neg	**Arenaria**
**WATC-D4**	**Triangle Cove**	**4/20/2022**	107764.4	9262.43	3	**0.09403**	Arenaria	Arenaria	neg		**Arenaria**	Arenaria	Arenaria	neg	**Arenaria**
**WATC-D5**	**Triangle Cove**	**4/20/2022**	172187.1	53639.3	3	**0.45247**	Arenaria	Arenaria	neg		**Arenaria**	Arenaria	Arenaria	neg	**Arenaria**
**WATC-D6**	**Triangle Cove**	**4/20/2022**	101475.7	1	0	**0**	Het	Het			**Het**	Arenaria	Arenaria		**Arenaria**
**WATC-D7**	**Triangle Cove**	**4/20/2022**	95448.04	1	0	**0**	Arenaria	Arenaria			**Arenaria**	Arenaria	Arenaria		**Arenaria**
**WATC-D8**	**Triangle Cove**	**4/20/2022**	254239	121849	3	**0.92037**	Arenaria	Arenaria	pos	Het	**Het**	Arenaria	Arenaria	neg	**Arenaria**
**WATC-D9**	**Triangle Cove**	**4/20/2022**	43436.08	11.8259	3	**2.72E-04**	Arenaria	Arenaria			**Arenaria**	Arenaria	Arenaria		**Arenaria**
**WATC-E1**	**Triangle Cove**	**4/20/2022**	31069.98	1	1	**0**	Arenaria	Arenaria			**Arenaria**	Arenaria	Arenaria		**Arenaria**
**WATC-E10**	**Triangle Cove**	**4/20/2022**	58312.37	13561.8	3	**0.30305**	Arenaria	Arenaria	neg		**Arenaria**	Arenaria	Arenaria	neg	**Arenaria**
**WATC-E11**	**Triangle Cove**	**4/20/2022**	64273.61	14142.1	3	**0.2821**	Het	Het	pos	Het	**Het**	Arenaria	Arenaria	neg	**Arenaria**
**WATC-E12**	**Triangle Cove**	**4/20/2022**	57220.62	1	0	**0**	Arenaria	Arenaria			**Arenaria**	Arenaria	Arenaria		**Arenaria**
**WATC-E2**	**Triangle Cove**	**4/20/2022**	80733.17	14.0683	3	**1.74E-04**	Arenaria	Arenaria			**Arenaria**	Arenaria	Arenaria		**Arenaria**

**WATC-E3**	**Triangle Cove**	**4/20/2022**	89724.92	1	0	**0**	Arenaria	Arenaria			**Arenaria**	Arenaria	Arenaria		**Arenaria**
**WATC-E4**	**Triangle Cove**	**4/20/2022**	46174.67	135.028	3	**0.00293**	Arenaria	Arenaria			**Arenaria**	Arenaria	Arenaria		**Arenaria**
**WATC-E5**	**Triangle Cove**	**4/20/2022**	35280.25	1	0	**0**	Arenaria	Arenaria			**Arenaria**	Arenaria	Arenaria		**Arenaria**
**WATC-E6**	**Triangle Cove**	**4/20/2022**	381983	135725	3	**0.55115**	Arenaria	Arenaria	neg		**Arenaria**	Arenaria	Arenaria	neg	**Arenaria**
**WATC-E7**	**Triangle Cove**	**4/20/2022**	48480.89	1	0	**0**	Arenaria	Arenaria			**Arenaria**	Arenaria	Arenaria		**Arenaria**
**WATC-E8**	**Triangle Cove**	**4/20/2022**	62797.03	1	0	**0**	Arenaria	Arenaria			**Arenaria**	Arenaria	Arenaria		**Arenaria**
**WATC-E9**	**Triangle Cove**	**4/20/2022**	29964.61	904.877	3	**0.03114**	Arenaria	Arenaria			**Arenaria**	Arenaria			**Arenaria**
**WATC-2A1**	**Triangle Cove**	**5/10/2023**	209137.9	125.623	3	**6.01E-04**	Arenaria				**Arenaria**	Arenaria			**Arenaria**
**WATC-2A10**	**Triangle Cove**	**5/10/2023**	241446.5	10.5215	3	**4.36E-05**	Arenaria				**Arenaria**	Arenaria			**Arenaria**
**WATC-2A11**	**Triangle Cove**	**5/10/2023**	54461.03	1	0	**0**	Japonica				**Japonica**	Arenaria			**Arenaria**
**WATC-2A12**	**Triangle Cove**	**5/10/2023**	5380.744	1	0	**0**	Het				**Het**	Arenaria			**Arenaria**
**WATC-2A2**	**Triangle Cove**	**5/10/2023**	43235.58	1	0	**0**	Arenaria				**Arenaria**	Arenaria			**Arenaria**
**WATC-2A3**	**Triangle Cove**	**5/10/2023**	47298.48	1	0	**0**	Arenaria				**Arenaria**	Arenaria			**Arenaria**
**WATC-2A4**	**Triangle Cove**	**5/10/2023**	92565.23	1	0	**0**	Arenaria				**Arenaria**	Arenaria			**Arenaria**
**WATC-2A5**	**Triangle Cove**	**5/10/2023**	36259.56	499.429	3	**0.01397**	Arenaria				**Arenaria**	Arenaria			**Arenaria**
**WATC-2A6**	**Triangle Cove**	**5/10/2023**	140473.7	176.171	3	**0.00126**	Arenaria				**Arenaria**	Arenaria			**Arenaria**
**WATC-2A7**	**Triangle Cove**	**5/10/2023**	40943.08	1	0	**0**	Arenaria				**Arenaria**	Arenaria			**Arenaria**
**WATC-2A8**	**Triangle Cove**	**5/10/2023**	56033.13	538.612	3	**0.00971**	Arenaria				**Arenaria**	Arenaria			**Arenaria**
**WATC-2A9**	**Triangle Cove**	**5/10/2023**	109640.9	6192.38	3	**0.05986**	Arenaria				**Arenaria**	Arenaria			**Arenaria**
**WATC-2B1**	**Triangle Cove**	**5/10/2023**	272373.5	137270	3	**1.01603**	Arenaria		neg	Arenaria	**Arenaria**	Arenaria		neg	**Arenaria**
**WATC-2B2**	**Triangle Cove**	**5/10/2023**	26231.87	1	0	**0**	Arenaria				**Arenaria**	Arenaria			**Arenaria**
**WATC-F1**	**Triangle Cove**	**5/10/2023**	78011.58	1	0	**0**	Arenaria				**Arenaria**	Arenaria			**Arenaria**
**WATC-F10**	**Triangle Cove**	**5/10/2023**	58555.71	250.143	3	**0.00429**	Het				**Het**	Arenaria			**Arenaria**
**WATC-F11**	**Triangle Cove**	**5/10/2023**	10028.15	1	0	**0**	Japonica				**Japonica**	Japonica			**Japonica**
**WATC-F12**	**Triangle Cove**	**5/10/2023**	100856	572.188	3	**0.00571**	Arenaria				**Arenaria**	Arenaria			**Arenaria**
**WATC-F2**	**Triangle Cove**	**5/10/2023**	186081.9	1	0	**0**	Arenaria				**Arenaria**	Arenaria			**Arenaria**
**WATC-F3**	**Triangle Cove**	**5/10/2023**	45205.82	1	0	**0**	Arenaria				**Arenaria**	Arenaria			**Arenaria**
**WATC-F4**	**Triangle Cove**	**5/10/2023**	5908.931	1	0	**0**	Arenaria				**Arenaria**	Arenaria			**Arenaria**
**WATC-F5**	**Triangle Cove**	**5/10/2023**	55210.07	236.017	3	**0.00429**	Het				**Het**	Arenaria			**Arenaria**
**WATC-F6**	**Triangle Cove**	**5/10/2023**	166737	1	0	**0**	Arenaria				**Arenaria**	Japonica			**Japonica**
**WATC-F7**	**Triangle Cove**	**5/10/2023**	50782.38	1.09868	1	**0**	Arenaria				**Arenaria**	Arenaria			**Arenaria**
**WATC-F8**	**Triangle Cove**	**5/10/2023**	121743.3	54289.8	3	**0.80485**	Arenaria		neg		**Arenaria**	Arenaria		neg	**Arenaria**
**WATC-F9**	**Triangle Cove**	**5/10/2023**	24136.77	1	0	**0**	Arenaria				**Arenaria**	Arenaria			**Arenaria**
**WATC-G1**	**Triangle Cove**	**5/10/2023**	57666.49	3.33523	2	**0**	Japonica				**Japonica**	Japonica			**Japonica**
**WATC-G10**	**Triangle Cove**	**5/10/2023**	79943.36	1	0	**0**	Het				**Het**	Arenaria			**Arenaria**
**WATC-G11**	**Triangle Cove**	**5/10/2023**	284954.7	1	0	**0**	Arenaria				**Arenaria**	Arenaria			**Arenaria**
**WATC-G12**	**Triangle Cove**	**5/10/2023**	126567.1	14016.3	3	**0.12453**	Het		pos	Japonica	**Japonica**	Inconclusive		pos	**Japonica**
**WATC-G2**	**Triangle Cove**	**5/10/2023**	23374.63	1	0	**0**	Arenaria				**Arenaria**	Arenaria			**Arenaria**
**WATC-G3**	**Triangle Cove**	**5/10/2023**	66438.4	3.21393	3	**4.84E-05**	Arenaria				**Arenaria**	Arenaria			**Arenaria**
**WATC-G4**	**Triangle Cove**	**5/10/2023**	81431.08	1	0	**0**	Arenaria				**Arenaria**	Arenaria			**Arenaria**
**WATC-G5**	**Triangle Cove**	**5/10/2023**	175394.5	1	0	**0**	Arenaria				**Arenaria**	Arenaria			**Arenaria**
**WATC-G6**	**Triangle Cove**	**5/10/2023**	7608.415	1	0	**0**	Het				**Het**	Arenaria			**Arenaria**
**WATC-G7**	**Triangle Cove**	**5/10/2023**	118667.5	968.091	3	**0.00823**	Het				**Het**	Arenaria			**Arenaria**
**WATC-G8**	**Triangle Cove**	**5/10/2023**	74265.64	1	0	**0**	Arenaria				**Arenaria**	Arenaria			**Arenaria**
**WATC-G9**	**Triangle Cove**	**5/10/2023**	113866.9	1	0	**0**	Japonica				**Japonica**	Arenaria			**Arenaria**
**WATC-H1**	**Triangle Cove**	**5/10/2023**	132407.4	1	0	**0**	Arenaria				**Arenaria**	Arenaria			**Arenaria**

**WATC-H10**	**Triangle Cove**	**5/10/2023**	93997.18	1	0	**0**	Arenaria		**Arenaria**	Arenaria		**Arenaria**
**WATC-H11**	**Triangle Cove**	**5/10/2023**	55197.03	1	0	**0**	Het		**Het**	Arenaria		**Arenaria**
**WATC-H12**	**Triangle Cove**	**5/10/2023**	6799.028	1	0	**0**	Japonica		**Japonica**	Japonica		**Japonica**
**WATC-H2**	**Triangle Cove**	**5/10/2023**	243500.1	692.114	3	**0.00285**	Arenaria		**Arenaria**	Arenaria		**Arenaria**
**WATC-H3**	**Triangle Cove**	**5/10/2023**	111932.1	21502.2	3	**0.23778**	Arenaria	neg	**Arenaria**	Arenaria	neg	**Arenaria**
**WATC-H4**	**Triangle Cove**	**5/10/2023**	66761.72	1	0	**0**	Arenaria		**Arenaria**	Arenaria		**Arenaria**
**WATC-H5**	**Triangle Cove**	**5/10/2023**	140556.6	1	0	**0**	Arenaria		**Arenaria**	Arenaria		**Arenaria**
**WATC-H6**	**Triangle Cove**	**5/10/2023**	73962.94	1	0	**0**	Het		**Het**	Arenaria		**Arenaria**
**WATC-H7**	**Triangle Cove**	**5/10/2023**	17415.29	1.05329	1	**0**	Het		**Het**	Arenaria		**Arenaria**
**WATC-H8**	**Triangle Cove**	**5/10/2023**	34269.06	1	0	**0**	Arenaria		**Arenaria**	Arenaria		**Arenaria**
**WATC-H9**	**Triangle Cove**	**5/10/2023**	9854.694	1	0	**0**	Japonica		**Japonica**	Arenaria		**Arenaria**
**WATC-2B10**	**Triangle Cove**	**7/8/2024**	35034.03	1	1	**0**	Arenaria		**Arenaria**	Arenaria		**Arenaria**
**WATC-2B11**	**Triangle Cove**	**7/8/2024**	49152.58	1	0	**0**	Arenaria		**Arenaria**	Arenaria		**Arenaria**
**WATC-2B12**	**Triangle Cove**	**7/8/2024**	56895.04	594.554	3	**0.01056**	Arenaria		**Arenaria**	Arenaria		**Arenaria**
**WATC-2B3**	**Triangle Cove**	**7/8/2024**	53391.56	1	0	**0**	Arenaria		**Arenaria**	Arenaria		**Arenaria**
**WATC-2B4**	**Triangle Cove**	**7/8/2024**	40489.58	1	0	**0**	Arenaria		**Arenaria**	Arenaria		**Arenaria**
**WATC-2B5**	**Triangle Cove**	**7/8/2024**	69895.43	2.59328	3	**3.71E-05**	Arenaria		**Arenaria**	Arenaria		**Arenaria**
**WATC-2B6**	**Triangle Cove**	**7/8/2024**	19371.23	87.6746	3	**0.00455**	Arenaria		**Arenaria**	Arenaria		**Arenaria**
**WATC-2B7**	**Triangle Cove**	**7/8/2024**	38591.98	30.5982	3	**7.93E-04**	Arenaria		**Arenaria**	Arenaria		**Arenaria**
**WATC-2B8**	**Triangle Cove**	**7/8/2024**	187293.2	85364.1	3	**0.83749**	Arenaria	neg	**Arenaria**	Arenaria	neg	**Arenaria**
**WATC-2B9**	**Triangle Cove**	**7/8/2024**	31402.91	6.06529	3	**1.93E-04**	Arenaria		**Arenaria**	Arenaria		**Arenaria**
**WATC-2C1**	**Triangle Cove**	**7/8/2024**	40932.21	1	0	**0**	Arenaria		**Arenaria**	Arenaria		**Arenaria**
**WATC-2C10**	**Triangle Cove**	**7/8/2024**	114401.8	72572.5	3	**1.73497**	Arenaria	neg	**Arenaria**	Arenaria	neg	**Arenaria**
**WATC-2C11**	**Triangle Cove**	**7/8/2024**	152100.5	66955	3	**0.78636**	Arenaria	neg	**Arenaria**	Arenaria	neg	**Arenaria**
**WATC-2C12**	**Triangle Cove**	**7/8/2024**	25387.37	27.1914	3	**0.00107**	Het		**Het**	Japonica		**Japonica**
**WATC-2C2**	**Triangle Cove**	**7/8/2024**	57051.49	1.8417	3	**3.23E-05**	Arenaria		**Arenaria**	Arenaria		**Arenaria**
**WATC-2C3**	**Triangle Cove**	**7/8/2024**	230000.4	88356.2	3	**0.62379**	Arenaria	neg	**Arenaria**	Arenaria	neg	**Arenaria**
**WATC-2C4**	**Triangle Cove**	**7/8/2024**	47513.37	28.7959	3	**6.06E-04**	Arenaria		**Arenaria**	Arenaria		**Arenaria**
**WATC-2C5**	**Triangle Cove**	**7/8/2024**	101608.7	31320.2	3	**0.4456**	Arenaria	neg	**Arenaria**	Arenaria	neg	**Arenaria**
**WATC-2C6**	**Triangle Cove**	**7/8/2024**	164043.2	66272.2	3	**0.67783**	Arenaria	neg	**Arenaria**	Arenaria	neg	**Arenaria**
**WATC-2C7**	**Triangle Cove**	**7/8/2024**	177738.8	74323.6	3	**0.71869**	Arenaria	neg	**Arenaria**	Arenaria	neg	**Arenaria**
**WATC-2C8**	**Triangle Cove**	**7/8/2024**	50348.7	346.696	3	**0.00693**	Arenaria		**Arenaria**	Arenaria		**Arenaria**
**WATC-2C9**	**Triangle Cove**	**7/8/2024**	185991.7	83722.7	3	**0.81865**	Arenaria	neg	**Arenaria**	Arenaria	neg	**Arenaria**
**WATC-2D1**	**Triangle Cove**	**7/8/2024**	15952.19	3.78629	3	**2.37E-04**	Arenaria		**Arenaria**	Arenaria		**Arenaria**
**WATC-2D2**	**Triangle Cove**	**7/8/2024**	23500.27	7.77408	3	**3.31E-04**	Het		**Het**	Japonica		**Japonica**
**WATC-2D3**	**Triangle Cove**	**7/8/2024**	238313.5	707.764	3	**0.00298**	Arenaria		**Arenaria**	Arenaria		**Arenaria**
**WATC-2D4**	**Triangle Cove**	**7/8/2024**	18579.23	8.44927	3	**4.55E-04**	Het		**Het**	Arenaria		**Arenaria**
**WATC-2D5**	**Triangle Cove**	**7/8/2024**	210555.8	50.7272	3	**2.41E-04**	Arenaria		**Arenaria**	Arenaria		**Arenaria**

Table S2. Primers used

Target	Forward		Reverse		Size	Reference
**Nuclear qPCR Primers**						
Steamer/N1N2	ClamLTR-F3	TTCAATCATTCAACGCATAACC	N1N2can-R3	TCGCTGAGAATTTTTCGGTGT	81 bp	(Giersch et al, 2022)
M. arenaria control	N1N2-F3	CCCAGGGCAAGAGGAATATGGT	N1N2-R1	GGATACTGCAAGCTTCTTGGAA	75 bp	(Giersch et al, 2022)
**Mitochondrial qPCR Primers**						
MarBTN-USA	MarBTN-mt8807F1	GGGCACATTAGAGTATGAATATg	MarBTN-mt8843R2	AAGCAGAAAAAGGCACTTGAa	84 bp	current manuscript
M. arenaria control	MarBTN-mt8807NORMF1	GGGGCACATTAGAGTATGAATAT	MarBTN-mt8843NORMR1	TAACCAAGCAGAAAAAGGCACTTGA	86 bp	current manuscript
M. japonica control	Mjp-mt8807NORMF1	TGGGCATATTAGGGTTTGAATAT	Mjp-mt8843NORMR1	TAGCCAAGCAGAAAATGGCACTTGT	86 bp	current manuscript
**Cytochrome b oxidase I**	LCO1490	GGTCAACAAATCATAAAGATATTGG	HCO2198	TAAACTTCAGGGTGACCAAAAAATCA	712 bp	(Folmer et al, 1994)
Optimized for *Mya spp*.	LCO1490Mar	CTAGGGCAAACCATAAAGATATHGG	HCO2198Mar	TAGACCTCTGGGTGTCCAAARAAYCA	712 bp	current manuscript
Specific for *M. japonica*	mtCOI-Mjp-F2	GACTGGTCTTAGTGTTC	mtCOI-Mjp-R2	AGTCAAAAGCATTGTTAGT	547 bp	current manuscript
***Steamer* insertion sites**	ClamLTR-F2	ACATGCACATTAAAAGTTATCG	IMA10c2R	taagtcagattgatgatacac	154 bp	(Metzger et al, 2015)
			IMA10c4R	tattttgacatttcttatgtttcc	170 bp	(Metzger et al, 2015)
			IMC8c6R	atgcttccattgcatcaaagt	142 bp	(Metzger et al, 2015)
			IMNYTCC9c7R	TGCATCAAAGTAGGAATCTG	132 bp	(Metzger et al, 2015)
			IMDL8c1R	aactccaatacccttcaatt	133 bp	(Arriagada et al, 2014)
			IMDL8c6R	agctgtctagattggaagtg	138 bp	(Arriagada et al, 2014)
			IMHL03c2R	attgtcccagattcacagat	121 bp	(Arriagada et al, 2014)
			IMA11c3R	tgcaggtcctattaaagtaaac	134 bp	(Metzger et al, 2015)
			IMA11c4R	aataggatgccaatatacttg	142 bp	(Metzger et al, 2015)
			IMC8c1R	cagcgaagaactgatggtga	149 bp	(Metzger et al, 2015)
			IMNYTCC9c2R	TACATAAATGATGTACATAAG	125 bp	(Metzger et al, 2015)
			Mar_sc0_USAin2-R	GGGCATTGAAATGTAGCAAA	167 bp	current manuscript
			IMHL03c3R	gtaggtcttatacatttgag	130 bp	(Arriagada et al, 2014)
			Mar_sc0_PEIin1-R	CGAGAACCATGTTCACTGTA	134 bp	current manuscript
			Mar_sc2_PEIin2-R	CTGAATGTGCCTGGTAAGTT	127 bp	current manuscript
			Mar_sc4_PEIin1-R	GTCATACCTGCTCAGTTTCT	142 bp	current manuscript
			Mar_sc5_PEIin1-R	TCTACGAAGTTTCCTGTGTG	124 bp	current manuscript
**Elongation Factor I**						
Optimized for *Mya spp*.	EF1-MarMjp-F1	CGATCTGGCAAGAAGCTGGA	EF1-MarMjp-R1	ATGTCTTGAAATGTGGACATGGG	334 bp	current manuscript
Specific for *M. japonica*	EF1-Mjp-F2	CTGTGAAGTCTGGGGAC	EF1-Mjp-R2	CCACATTTTCCTTCTATGA	247 bp	current manuscript

Table S3. Quantification of MarBTN in eDNA from seawater collected from multiple sites in Puget Sound, WA, USA

Site	ID	Map	Latitude	Longitude	Date	Sample method	Preservation method	DNA extraction	MarBTN copies per mL of seawater	*M. arenaria* copies per mL of seawater	*M. japonica* copies per mL of seawater	MarBTN copies per reaction*	*M. arenaria* copies per reaction*	*M. japonica* copies per reaction*	*M. arenaria* fraction**
Crandall Spit	CS	Y	48.490611	-122.580611	6/15/2023	Polypro bottle (at shore)	Water sample frozen	Qiagen Powersoil Pro	0	2.151284	61.70115344	1	21.51284	617.0115344	0.033691494
False Bay	FB	Y	48.488141	-123.068496	7/19/2023	Polypro bottle (at shore)	Water sample frozen	Qiagen Powersoil Pro	0	0	0.204551962	1	1	2.045519617	Und.
Similk Bay	SK	Y	48.44217	-122.571981	3/15/2024	Niskin (near bottom)	Filter dessicated	Qiagen Powersoil Pro	0	5.897212325	1.267365122	1	58.97212325	12.67365122	0.823106787
Cornet Bay	CB	Y	48.397953	-122.634201	3/15/2024	Niskin (near bottom)	Filter dessicated	Qiagen Powersoil Pro	0.143484773	0.272036122	0.110209526	1.434847726	2.72036122	1.10209526	0.538410446
Marthas Bay	MA	Y	48.374039	-122.525592	3/15/2024	Niskin (near bottom)	Filter dessicated	Qiagen Powersoil Pro	1.472020589	1.57354792	0	14.72020589	15.7354792	1	1
Dugualla Bay	DB	Y	48.357082	-122.592269	3/15/2024	Niskin (near bottom)	Filter dessicated	Qiagen Powersoil Pro	0	0	0	1	1	1	Und.
S Dugualla State Park	SD	Y	48.336776	-122.539458	3/15/2024	Niskin (near bottom)	Filter dessicated	Qiagen Powersoil Pro	0.651161705	0.891520079	0	6.511617051	8.91520079	1	1
Hall Slough	HS	Y	48.335727	-122.43895	3/15/2024	Niskin (near bottom)	Filter dessicated	Qiagen Powersoil Pro	22.3666303	77.50515001	0.296052477	223.666303	775.0515001	2.960524771	0.994659425
Wiley Slough	WS	Y	48.317002	-122.411458	3/15/2024	Niskin (near bottom)	Filter dessicated	Qiagen Powersoil Pro	51.14842699	62.16888224	0.101086185	511.4842699	621.6888224	1.010861847	0.990910776
Strawberry Point	SP	Y	48.300254	-122.50371	3/15/2024	Niskin (near bottom)	Filter dessicated	Qiagen Powersoil Pro	1.106598144	1.948627954	0	11.06598144	19.48627954	1	1
Isohis Slough	IS	Y	48.297092	-122.401538	3/15/2024	Niskin (near bottom)	Filter dessicated	Qiagen Powersoil Pro	10.50153037	9.65672955	0.124218146	105.0153037	96.5672955	1.24218146	1.172385696
Crescent Harbor	CN	Y	48.287962	-122.597766	3/15/2024	Niskin (near bottom)	Filter dessicated	Qiagen Powersoil Pro	0	0.296071794	0	1	2.960717943	1	1
Oak Harbor	OK	Y	48.277496	-122.651915	3/15/2024	Niskin (near bottom)	Filter dessicated	Qiagen Powersoil Pro	0.115187817	0.182829264	0.106003229	1.151878171	1.828292635	1.060032288	0.389539422
Eastern Bank	P22-B	N	48.2717	-123.0189	7/9/2024	Niskin (near bottom)	Filter in Longmire's	Modified CTAB	0	0	0	1	1	1	Und.
Eastern Bank	P22-M	N	48.2717	-123.0189	7/9/2024	Niskin (middle)	Filter in Longmire's	Modified CTAB	0	0	0	1	1	1	Und.
Eastern Bank	P22-S	Y	48.2717	-123.0189	7/9/2024	Niskin (surface)	Filter in Longmire's	Modified CTAB	0	0	0	1	1	1	Und.
Big Ditch Trail	BD	Y	48.271592	-122.40523	3/15/2024	Niskin (near bottom)	Filter dessicated	Qiagen Powersoil Pro	35.21840549	32.23771803	0.169607756	352.1840549	322.3771803	1.696077559	1.060335449
Big Ditch Trail	BD	N	48.271592	-122.40523	5/8/2025	Niskin (near bottom)	Water sample frozen	Qiagen Powersoil Pro	372.0094971	2853.015126	167.2716208	3720.094971	28530.15126	1672.716208	0.936837572
Big Ditch Trail	BD	N	48.271592	-122.40523	5/8/2025	Niskin (near bottom)	Filter dessicated	Qiagen Powersoil Pro	980.9675022	4038.907943	141.9011868	9809.675022	40389.07943	1419.011868	0.955653684
Big Ditch Trail	BD	N	48.271592	-122.40523	5/8/2025	Niskin (near bottom)	Filter in Longmire's	Modified CTAB	272.4498101	2410.387674	50.82375505	1362.249051	12051.93837	254.1187753	0.976779675
English Boom County Park	EB	Y	48.267127	-122.440248	3/15/2024	Niskin (near bottom)	Filter dessicated	Qiagen Powersoil Pro	20.00085093	18.72818061	0.129606729	200.0085093	187.2818061	1.296067291	1.113385406
Utsalady Bay	UD	Y	48.258013	-122.50371	3/15/2024	Niskin (near bottom)	Filter dessicated	Qiagen Powersoil Pro	0.167685604	0.253896725	0	1.676856041	2.538967252	1	1
Skagit Bay	P4-B	N	48.2422	-122.5533	7/8/2024	Niskin (near bottom)	Filter in Longmire's	Modified CTAB	0	0.285877045	0	1	1.429385225	1	1
Skagit Bay	P4-M	N	48.2422	-122.5533	7/8/2024	Niskin (middle)	Filter in Longmire's	Modified CTAB	0	0.241111469	0	1	1.205557346	1	1
Skagit Bay	P4-S	Y	48.2422	-122.5533	7/8/2024	Niskin (surface)	Filter in Longmire's	Modified CTAB	36.92962138	248.7857422	0.169056869	184.6481069	1243.928711	1.690568686	0.999202657
Penn Cove	PC	Y	48.239139	-122.679333	12/11/2023	Polypro bottle (at shore)	Water sample frozen	Qiagen Powersoil Pro	0.568017567	0.8284405	1.010743093	5.680175667	8.284405	10.10743093	0.204869331
Kettles Trailhead (West Penn)	KT	Y	48.230453	-122.732207	3/15/2024	Niskin (near bottom)	Filter dessicated	Qiagen Powersoil Pro	0	0	0	1	1	1	Und.
West Penn	WP	Y	48.228444	-122.732139	6/7/2024	Polypro bottle (at shore)	Water sample frozen	Qiagen Powersoil Pro	0	0.2090664	0.854128064	1	2.090664	8.54128064	0.19663985
Livingston Bay	LB	Y	48.22809	-122.439416	4/9/2024	Polypro bottle (at shore)	Water sample frozen	Qiagen Powersoil Pro	22.24035975	32.26881341	1.013161242	222.4035975	322.6881341	10.13161242	0.908241571
Long Point	LP	Y	48.226974	-122.660029	3/15/2024	Niskin (near bottom)	Filter dessicated	Qiagen Powersoil Pro	0	0.162671999	0.173341719	1	1.626719991	1.733417193	0.484123088
Triangle Cove	TC	Y	48.199121	-122.473384	8/27/2024	Polypro bottle (at shore)	Water sample frozen	Qiagen Powersoil Pro	735.8227376	989.3119846	8.986304601	7358.227376	9893.119846	89.86304601	0.96576327
Saratoga Passage	ST	Y	48.184516	-122.581072	3/15/2024	Niskin (near bottom)	Filter dessicated	Qiagen Powersoil Pro	0	0	0	1	1	1	Und.
Cavalero Boat Launch	BL	Y	48.174634	-122.477024	4/9/2024	Polypro bottle (at shore)	Water sample frozen	Qiagen Powersoil Pro	13.9644377	21.93580805	1.266448964	139.644377	219.3580805	12.66448964	0.862906069
Hidden Beach	HB	Y	48.128008	-122.559879	3/15/2024	Niskin (near bottom)	Filter dessicated	Qiagen Powersoil Pro	0	0	0	1	1	1	Und.
Camano Island State Park	CP	Y	48.121015	-122.491271	4/9/2024	Polypro bottle (at shore)	Water sample frozen	Qiagen Powersoil Pro	0	0.293064499	0.275742634	1	2.930644989	2.757426341	0.515226483
Tillicum Beach	TL	Y	48.103682	-122.399601	4/9/2024	Polypro bottle (at shore)	Water sample frozen	Qiagen Powersoil Pro	0.674852943	10.79817859	0.596902588	6.748529434	107.9817859	5.969025877	0.944319974
Mabana Beach	MB	Y	48.092998	-122.416233	4/9/2024	Polypro bottle (at shore)	Water sample frozen	Qiagen Powersoil Pro	0	1.230466101	1.674309616	1	12.30466101	16.74309616	0.42360107
TU-2	TU-2	Y	48.057059	-122.274826	3/29/2024	Niskin (near bottom)	Water sample frozen	Qiagen Powersoil Pro	3.134735415	3.250330586	0.199617615	31.34735415	32.50330586	1.99617615	0.366721073
TU-4	TU-4	Y	48.053889	-122.285297	3/29/2024	Niskin (near bottom)	Water sample frozen	Qiagen Powersoil Pro	2.015399653	2.914565696	0.245055848	20.15399653	29.14565696	2.450558477	0.785831883
TU-3	TU-3	Y	48.053545	-122.273967	3/29/2024	Niskin (near bottom)	Water sample frozen	Qiagen Powersoil Pro	1.549628766	2.150296585	0	15.49628766	21.50296585	1	1
Honeymoon Bay	HM	Y	48.053099	-122.549246	4/15/2024	Polypro bottle (at shore)	Water sample frozen	Qiagen Powersoil Pro	0	479.9183458	413.3884928	1	4799.183458	4133.884928	0.537237962
Sequim Bay	SB	Y	48.041583	-123.026833	4/10/2023	Polypro bottle (at shore)	Water sample frozen	Qiagen Powersoil Pro	0	1.197372993	15.42078951	1	11.97372993	154.2078951	0.072052069
South Whidbey Harbor	SW	Y	48.039403	-122.403646	4/15/2024	Polypro bottle (at shore)	Water sample frozen	Qiagen Powersoil Pro	0.679008259	3.54605448	1.634420013	6.790082587	35.4605448	16.34420013	0.636913857
TU-1	TU-1	Y	48.030361	-122.239893	3/29/2024	Niskin (near bottom)	Water sample frozen	Qiagen Powersoil Pro	0	0.138460267	1.184344779	1	1.384602673	11.84344779	0.104671711
Gedney Island	P1-B	N	48.0165	-122.3042	7/8/2024	Niskin (near bottom)	Filter in Longmire's	Modified CTAB	0	0	0	1	1	1	Und.
Gedney Island	P1-S	Y	48.0165	-122.3042	7/8/2024	Niskin (surface)	Filter in Longmire's	Modified CTAB	0.212557316	254.1841064	0.228584121	1.062786579	1270.920532	2.285841207	0.999100771
Mutiny Bay	P7-B	N	47.9835	-122.6201	7/9/2024	Niskin (near bottom)	Filter in Longmire's	Modified CTAB	0	0	0	1	1	1	Und.
Mutiny Bay	P7-M	Y	47.9835	-122.6201	7/9/2024	Niskin (middle)	Filter in Longmire's	Modified CTAB	0	0	0.358073616	1	1	3.58073616	Und.
Mutiny Bay	P7-S	N	47.9835	-122.6201	7/9/2024	Niskin (surface)	Filter in Longmire's	Modified CTAB	0	0	0	1	1	1	Und.
Clinton Ferry	CL	Y	47.975502	-122.351307	4/15/2024	Polypro bottle (at shore)	Water sample frozen	Qiagen Powersoil Pro	0.437924814	0.482712213	0.205993651	4.379248142	4.827122132	2.05993651	0.17859164
Hood Head	P8-B	N	47.8967	-122.6053	7/9/2024	Niskin (near bottom)	Filter in Longmire's	Modified CTAB	0	0.204625622	0	1	1.023128112	1	1
Hood Head	P8-M	Y	47.8967	-122.6053	7/9/2024	Niskin (middle)	Filter in Longmire's	Modified CTAB	0	0.225696262	0	1	1.128481309	1	1
Hood Head	P8-S	N	47.8967	-122.6053	7/9/2024	Niskin (surface)	Filter in Longmire's	Modified CTAB	0	0	0	1	1	1	Und.
Hood Canal Sill	P10-B	Y	47.8001	-122.7198	7/10/2024	Niskin (near bottom)	Filter in Longmire's	Modified CTAB	0	0.486595265	0	1	2.432976325	1	1
Hood Canal Sill	P10-M	N	47.8001	-122.7198	7/10/2024	Niskin (middle)	Filter in Longmire's	Modified CTAB	0	0.338089577	0	1	1.690447887	1	1
Hood Canal Sill	P10-S	N	47.8001	-122.7198	7/10/2024	Niskin (surface)	Filter in Longmire's	Modified CTAB	0	0	0	1	1	1	Und.

North of West Pt	P28-B	N	47.7034	-122.4544	7/8/2024	Niskin (near bottom)	Filter in Longmire's	Modified CTAB	0	0	0	1	1	1	Und.
North of West Pt	P28-M	N	47.7034	-122.4544	7/8/2024	Niskin (middle)	Filter in Longmire's	Modified CTAB	0	0	0	1	1	1	Und.
North of West Pt	P28-S	Y	47.7034	-122.4544	7/8/2024	Niskin (surface)	Filter in Longmire's	Modified CTAB	0	0.392427421	0	1	1.962137103	1	1
Hood Point	P14-B	N	47.6068	-122.9399	7/10/2024	Niskin (near bottom)	Filter in Longmire's	Modified CTAB	0	0.247791433	0	1	1.238957167	1	1
Hood Point	P14-M	N	47.6068	-122.9399	7/10/2024	Niskin (middle)	Filter in Longmire's	Modified CTAB	0	0	0	1	1	1	Und.
Hood Point	P14-S	Y	47.6068	-122.9399	7/10/2024	Niskin (surface)	Filter in Longmire's	Modified CTAB	0	0	0.304995747	1	1	3.049957474	Und.
South of Alki Pt	P29-B	N	47.5568	-122.4433	7/11/2024	Niskin (near bottom)	Filter in Longmire's	Modified CTAB	0	0	0	1	1	1	Und.
South of Alki Pt	P29-M	N	47.5568	-122.4433	7/11/2024	Niskin (middle)	Filter in Longmire's	Modified CTAB	0	0.391761204	0	1	1.958806018	1	1
South of Alki Pt	P29-S	Y	47.5568	-122.4433	7/11/2024	Niskin (surface)	Filter in Longmire's	Modified CTAB	0	0.427675978	0	1	2.138379892	1	1
Hoodsport	P12-B	N	47.4253	-123.1083	7/10/2024	Niskin (near bottom)	Filter in Longmire's	Modified CTAB	0	0	0	1	1	1	Und.
Hoodsport	P12-M	Y	47.4253	-123.1083	7/10/2024	Niskin (middle)	Filter in Longmire's	Modified CTAB	0	0.257477283	0	1	1.287386417	1	1
Hoodsport	P12-S	N	47.4253	-123.1083	7/10/2024	Niskin (surface)	Filter in Longmire's	Modified CTAB	0	0	0	1	1	1	Und.
The Great Bend	P11-B	Y	47.3712	-123.1329	7/10/2024	Niskin (near bottom)	Filter in Longmire's	Modified CTAB	0	1.223783223	0	1	6.118916114	1	1
The Great Bend	P11-M	N	47.3712	-123.1329	7/10/2024	Niskin (middle)	Filter in Longmire's	Modified CTAB	0	0	0	1	1	1	Und.
The Great Bend	P11-S	N	47.3712	-123.1329	7/10/2024	Niskin (surface)	Filter in Longmire's	Modified CTAB	0	0	0	1	1	1	Und.
Sisters Point	P402-B	N	47.3567	-123.0233	7/10/2024	Niskin (near bottom)	Filter in Longmire's	Modified CTAB	0	0	0	1	1	1	Und.
Sisters Point	P402-M	Y	47.3567	-123.0233	7/10/2024	Niskin (middle)	Filter in Longmire's	Modified CTAB	0	0.3179039	0	1	1.589519501	1	1
Sisters Point	P402-S	N	47.3567	-123.0233	7/10/2024	Niskin (surface)	Filter in Longmire's	Modified CTAB	0	0	0	1	1	1	Und.
Dash Point	DP	Y	47.320694	-122.413694	8/2/2023	Polypro bottle (at shore)	Water sample frozen	Qiagen Powersoil Pro	0	2.845451667	3.020234489	1	28.45451667	30.20234489	0.485101247
Carr Inlet	P38-B	N	47.2766	-122.7082	7/12/2024	Niskin (near bottom)	Filter in Longmire's	Modified CTAB	0	0	0	1	1	1	Und.
Carr Inlet	P38-M	Y	47.2766	-122.7082	7/12/2024	Niskin (middle)	Filter in Longmire's	Modified CTAB	0	1.176017793	0	1	5.880088965	1	1
Carr Inlet	P38-S	N	47.2766	-122.7082	7/12/2024	Niskin (surface)	Filter in Longmire's	Modified CTAB	0	0	0	1	1	1	Und.
Devils Head	P36-B	N	47.1684	-122.7865	7/12/2024	Niskin (near bottom)	Filter in Longmire's	Modified CTAB	0	0	0	1	1	1	Und.
Devils Head	P36-M	N	47.1684	-122.7865	7/12/2024	Niskin (middle)	Filter in Longmire's	Modified CTAB	0	0	0	1	1	1	Und.
Devils Head	P36-S	Y	47.1684	-122.7865	7/12/2024	Niskin (surface)	Filter in Longmire's	Modified CTAB	0	0.325552289	0	1	1.627761443	1	1

*Limit of Detection for qPCR assay was determined to be 1 copy per reaction. Samples with undetectible amplification are listed with "1" in copies per reaction and "0" for copier per mL seawater.

**Calculated using copies per ml seawater, subtracting MarBTN copies from total *M. arenaria* copies: (*M. arenaria* - MarBTN)/(*M. arenaria* - MarBTN + *M. japonica*)

Und. = Undetectible

Text S1. Environmental DNA extraction protocol using Qiagen DNeasy PowerSoil Pro and full reagent list

Developed for detection of MarBTN and species-specific eDNA from seawater (for Weinandt and Child, et al), with input from Tim O'Donnell (Gloucester Marine Genomics Institute).

Note, the Qiagen PowerSoil kit is used instead of the PowerWater kit, as we found that PCR inhibitors contaminated extractions when using the PowerWater kit with seawater samples. We have found the following protocol leads to high yields of eDNA with minimal PCR inhibitors.

Steps:

1. Water should be kept on ice and filtered as soon as possible after collection. If water cannot be filtered within ~6 hrs, water can be frozen at -30°C before thawing and filtering (this can lead to loss of yield of up to ~½). Filter water using a 0.45 µm, 47 mm cellulose nitrate membrane, using an autoclaved benchtop glass vacuum filtration apparatus or a Single Use Analytical Filter Funnel.
2. Filter cutting and storage (proceed with step 3a OR 3b):
    a. If a -80°C freezer is available at the filtration site: Filters are immediately folded in half using sterile forceps over a sterile petri dish. Cut around the edge with sterile scissors to remove any filter without sample. Cut the filter into three strips, and carefully guide the strips into the tube with forceps. When the strip is halfway into the tube, cut it in half, letting the lower half fall into the PowerBead Pro Tube (provided in kit), and then place the upper half in alongside it. Store tube with filter at -80°C, until ready to proceed to extraction (starting with step 3).

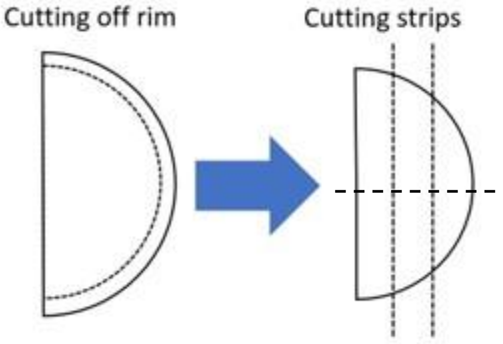

(Diagram from Tim O'Donnell, GMGI)

    b. If -80°C is not immediately accessible: filters are placed into sterile coin envelopes, and subsequently sealed in a mylar ziplock bag with a silica gel desiccant packet and held on ice for short-term preservation. Filters can be stored

in the bags for longer periods of time at -80°C. When ready to extract, remove filter from bag with sterile forceps over a sterile petri dish and cut as above, placing cut pieces in the PowerBead Pro Tube. Proceed immediately to step 3.

3. Add 800 µL Solution CD1 to PowerBead Pro Tube containing the filter.
4. Briefly vortex to mix.
5. Horizontally vortex at max speed for 10 min. We use a Vortex Adapter for 24 (1.5-2.0 ml) tubes from Qiagen.
    a. If doing >12 preps in an adaptor simultaneously, increase vortex time by 5-10 min
6. Centrifuge the PowerBead Pro Tube at 15,000 × g for 1 min.
7. Transfer 500-600 µL supernatant to a clean 2 mL tube (provided in kit).
8. Add 200 µL Solution CD2, vortex 5 sec.
9. Centrifuge at 15,000 × g for 1 min at room temperature.
10. Transfer up to 700 µ L supernatant to a clean 2 mL tube (provided in kit).
11. Add 600 µL Solution CD3 and vortex for 5 sec.
12. Load 650 µL lysate onto an MB Spin Column (provided in kit).
13. Centrifuge at 15,000 × g for 1 min.
14. Discard flow-through.
15. Repeat steps 11-13 until all lysate has passed through the MB Spin Column (to maximize DNA content).
16. Carefully place the MB Spin Column into a clean 2 mL Collection Tube (provided) - avoid splashing any flow-through onto the MB Spin column!
17. Add 500 µL of Solution EA to the MB Spin Column.
18. Centrifuge at 15,000 × g for 1 min.
19. Discard flow-through.
20. Place MB Spin Column back into same 2 mL Collection Tube (provided).
21. Add 500 µL Solution C5 to MB Spin Column.
22. Centrifuge at 15,000 × g for 1 min.
23. Discard flow-through.
24. Place MB Spin Column into a new 2 mL Collection Tube (provided).

1. Centrifuge up to 16,000 × g for 2 min.
2. Carefully place MB Spin Column into a new 1.5 mL Elution Tube (provided).
3. Add 100 µL Solution C6 to the center of the white filter membrane - wait at least 1 min.
4. Centrifuge at 15,000 × g for 1 min.
5. Discard spin column.

Store in -30°C long-term (C6/EB does not contain EDTA). Ideally, samples are not left at 4°C longer than 24-48 hours.

Reagents and Supplies	Supplier	Catalogue Number
MilliporeSigma™ Glass Vacuum Filter Holder, 47mm, Coarse-frit glass filter support	Fisher Scientific	XX1014700
PYREX™ Filtering Flasks with Tubulation, 1L	Fisher Scientific	10-180F
Cytiva Whatman™ Vacu-Guard Filter	Fisher Scientific	09-744-76
Nalgene™ Single Use Analytical Filter Funnels, 250 mL, 0.45um, white with black grid	Fisher Scientific	09-740-30K
Cytiva Whatman™ Type WCN Cellulose Nitrate Membranes, 0.45 um, 47 mm	Fisher Scientific	09-905-17
Exel 26G 0.5 inch sterile needle	Fisher Scientific	14-840-83
Fisherbrand™ Sterile Syringes for Single Use, 3 mL, luer-lock	Fisher Scientific	14-955-457
Fisherbrand™ 96-Well, Cell Culture-Treated, Flat-Bottom Microplate	Fisher Scientific	FB012931
Olympus 1.7ml Microtubes, Assorted	Genesee Scientific	24-282A
Monarch genomic DNA purification kit	New England Biolabs	T3010
NotI-HF	New England Biolabs	R3189S
Qubit™ 4 Fluorometer	ThermoFisher Scientific	Q33238
Applied Biosystems™ PowerUp™ SYBR™ Green Master Mix for qPCR	Fisher Scientific	A25743
Applied Biosystems™ StepOnePlus™ Real-Time PCR System	Fisher Scientific	43-766-00
Fisherbrand™ Graduated Polypropylene Laboratory Bottles, 500 mL	Fisher Scientific	03-405-33
DNeasy PowerSoil Pro Kit	Qiagen	47016
#1 Coin Envelopes, 2 1/4" x 3 1/2", Kraft paper	Amazon (Eupako)	N/A
Double-sided mylar metallic foil flat packaging zipper seal bags	Amazon (QQ Studio)	N/A
Dry&Dry Silica gel desiccant packet	Amazon (Dry&Dry)	N/A
Eppendorf® Centrifuge 5425 R	MilliporeSigma	EP5406000445
Applied Biosystems™ MiniAmp™ Plus Thermal Cycler	ThermoFisher Scientific	A37835
Apex Ultra pure, sterile, molecular biology grade water	Genesee Scientific	18-194
Apex Bioresearch Products 18-149 Tris-HCl, Molecular/Proteomic Grade	Genesee Scientific	18-149
Ethylenediaminetetraacetic Acid, Di Na Salt Dihydr. (Crystalline Powd./Electrophor.), Fisher BioReagents™	Fisher Scientific	BP120-500
Sodium Chloride (Crystalline/Biological, Certified), Fisher Chemical™	Fisher Scientific	S671-500
Sodium dodecyl sulfate solution	MilliporeSigma	7990-OP
GenClone 25-227, Vacuum Filter Systems, 500ml PES Membrane, 0.22µm, Sterile, 12 Systems/Unit	Genesee Scientific	25-227
Phenol:Chloroform:Isoamyl Alcohol 25:24:1 Saturated with 10 mM Tris, pH 8.0, 1 mM EDTA	Sigma Aldrich	P2069-100ML
Chloroform Isoamyl Alcohol Mixture BioUltra, 24:1, ≥99.5% (chloroform + isoamyl alcohol, GC), MilliporeSigma™ Supelco™	Fisher Scientific	11-101-6907
Olympus 2.0ml Microtubes, Clear	Genesee Scientific	24-282A
Proteinase K, Molecular Biology Grade	New England Biolabs	P8107S
MultiTherm™ shaker with heating	MilliporeSigma	Z755753
PowerBead Pro Tubes (2 ml)	Qiagen	19301
2-Propanol, Molecular Biology Grade, Fisher BioReagents™	Fisher Scientific	BP2618500
Invitrogen™ GlycoBlue™ Coprecipitant (15 mg/mL)	Fisher Scientific	AM9516
USA Scientific Inc Vortex-Genie 2	Fisher Scientific	NC9864336
Vortex Adapter for 24 (1.5-2.0 ml) tubes	Qiagen	13000-V1-24
Thermo Scientific™ NanoDrop™ 2000/2000c Spectrophotometers	Fisher Scientific	ND2000
Invitrogen™ Zero Blunt™ TOPO™ PCR Cloning Kit for Sequencing, without competent cells	Fisher Scientific	45-003-1

Applied Biosystems™ MicroAmp™ Fast Optical 96-Well Reaction Plate, 0.1mL	Fisher Scientific	43-469-07
Leica Microsystems DMi1 Manual Inverted Microscope	Fisher Scientific	01-672-695
Olympus Plastics 27-125U, 0.2ml 8-Strip PCR Tubes, Flex Free Individual Attached Flat Caps, 120 Strips/Unit	Genesee Scientific	27-125U
Q5 Hot Start High-Fidelity DNA Polymerase	New England Biolabs	M0493L
Apex Bioresearch Products 42-411 Apex dNTP Mix, 1 x 20µmol, 10mM each, 40mM Total, 1 x 500µl/Unit	Genesee Scientific	42-411
Apex Bioresearch Products 20-102GP Apex General Purpose Agarose, Ultra Pure, 500g/Unit	Genesee Scientific	20-102GP
Apex Bioresearch Products 20-276 EtBr Dropper Bottle, 10ml, Ethidium Bromide, 0.625mg/ml, 1 Bottle/Unit	Genesee Scientific	20-276
Apex Bioresearch Products 20-193 TAE 50X Liquid Concentrate, Ultra Pure Grade Solution, 1.6 Liters/Unit	Genesee Scientific	20-193
Guanosine	MilliporeSigma	G6752-100G
Gel Loading Dye, Purple (6X)	New England Biolabs	B7024S
GeneRuler 1 kb Plus DNA Ladder, ready-to-use	ThermoFisher Scientific	SM1333
Thermo Scientific™ Owl™ EasyCast™ B1 Mini Gel Electrophoresis Systems	Fisher Scientific	OW-B1-BP
Thermo Scientific™ Owl™ EC300XL2 Compact Power Supply	Fisher Scientific	FBEC300XL
